# Impact of diets high in trans-fatty acids on cardiovascular diseases in adults aged 55 and older: insights from the Global Burden of Disease 2021 data

**DOI:** 10.3389/fnut.2026.1681274

**Published:** 2026-03-06

**Authors:** Jishi Ye, Yu Liu, Juan Ren, Ruolan Wu, Jingli Chen, Rong Xiang, Yifan Jia

**Affiliations:** 1 Department of Pain, Renmin Hospital of Wuhan University, Wuhan, Hubei, China; 2 Department of Otolaryngology-Head and Neck Surgery, Renmin Hospital of Wuhan University, Wuhan, Hubei, China; 3 Department of Intensive Care Unit, The Central Hospital of Wuhan, Tongji Medical College, Huazhong University of Science and Technology, Wuhan, Hubei, China; 4 Department of Respiratory Medicine, General Hospital of Central Theater Command, Wuhan, China; 5 Department of Anesthesiology, The Central Hospital of Wuhan, Tongji Medical College, Huazhong University of Science and Technology, Wuhan, Hubei, China

**Keywords:** cardiovascular disease, Global Burden of Disease, health inequality, high trans-fatty acid, socioeconomic disparities

## Abstract

**Background:**

High trans-fatty acid (TFA) intake is a major modifiable risk factor for cardiovascular disease (CVD), especially in older adults. This study aimed to assess global trends and health inequalities in CVD burden attributable to high TFA intake from 1990 to 2021 and project future patterns through 2036.

**Methods:**

Using data from the Global Burden of Disease (GBD) Study 2021, we analyzed age-standardized mortality rates (ASMR), disability-adjusted life years (ASDR), and inequality indicators across 204 countries and territories. Age-Period-Cohort (APC) models and Bayesian projections were applied to estimate future trends.

**Results:**

Globally, ASMR and ASDR attributable to high TFA intake declined by 69 and 68%, respectively, from 1990 to 2021. The most significant reductions were observed in high-SDI regions, where comprehensive TFA bans and public health policies were implemented. In contrast, the absolute burden remains high in low- and middle-SDI countries due to limited policy enforcement and dietary interventions. Socioeconomic inequalities narrowed over time, but vulnerable populations still face elevated risks. Projections indicate a continued global decline in CVD burden attributable to TFA through 2036, though widening uncertainties reflect demographic and policy challenges.

**Conclusion:**

While global progress in reducing TFA-related CVD burden is evident, persistent disparities and emerging risks in low-resource settings underscore the need for global elimination of industrial TFA, strengthened health systems, and targeted strategies to protect high-risk groups.

## Highlights

- This study provides the first global assessment of long-term trends and socioeconomic inequalities in CVD burden attributable to high trans-fatty acid intake among adults aged 55 and older.
- Significant declines in CVD mortality and DALYs were observed globally, but low- and middle-income regions still face high burdens and persistent health disparities.
- Projections suggest continued global progress, yet rising uncertainties highlight the urgent need for coordinated policies to eliminate industrial TFA, especially in vulnerable populations.

## Introduction

Cardiovascular diseases (CVD), primarily including ischemic heart disease (IHD) and stroke, are the leading causes of death and disability worldwide, posing a significant burden on public health and healthcare systems (1, 2). The pathogenesis of CVD is characterized by endothelial dysfunction and chronic vascular wall inflammation, which gradually progress to atherosclerotic lesions and ultimately result in myocardial infarction and stroke (3, 4). With the global increase in major risk factors such as population aging, obesity, and diabetes, the burden of CVD has been rising markedly in both industrialized and developing countries (5–7). It is estimated that between 1990 and 2019, the global prevalence of CVD nearly doubled, and this trend is likely to continue in the coming years (6). Among these, adults aged 55 years and older—due to cumulative exposure to risk factors and age-related physiological decline—are at particularly high risk for CVD incidence and mortality (8).

Diet is a key modifiable risk factor for CVD, and high intake of trans-fatty acids (TFA) has drawn increasing attention due to its role in dyslipidemia, inflammatory responses, and promotion of atherosclerosis (9, 10). TFA are mainly found in partially hydrogenated vegetable oils, margarine, processed baked goods, and fried foods. Although some high-income countries have implemented strict regulations to limit TFA content, intake levels remain elevated in many parts of the world, particularly in low- and middle-income countries (11, 12). Numerous epidemiological studies have confirmed that TFA intake is significantly associated with coronary heart disease, stroke, and other CVDs (13). Older adults are particularly vulnerable to the long-term harmful effects of TFA, with higher incidence, mortality, and long-term disability risks observed in this population (14, 15). Therefore, evaluating the CVD burden attributable to high TFA intake among individuals aged 55 years and older is essential for formulating targeted dietary interventions, optimizing resource allocation, and reducing healthcare burdens.

The Global Burden of Disease (GBD) study provides valuable data and methodologies for quantifying disease burdens attributable to various risk factors. The GBD database systematically assesses the health burden of 369 diseases and injuries and 87 risk factors across 204 countries and territories from 1990 to 2021 (16). However, a comprehensive analysis focusing on the CVD mortality and disability-adjusted life years (DALYs) burden attributable to high TFA intake among adults aged 55 and above is still lacking. Based on GBD 2021 data, this study employs an age–period–cohort (APC) model to comprehensively analyze the trends in CVD burden attributable to TFA intake at global, regional, and national levels from 1990 to 2021. It also examines differences by sociodemographic index (SDI), sex, and geographic region, aiming to provide data support and policy implications for CVD prevention and control in older populations worldwide.

## Methods

The Global Burden of Disease Study 2021 (GBD 2021), led by the Institute for Health Metrics and Evaluation (IHME), is a large-scale international collaborative project. Based on sustained cooperation across countries, the study systematically evaluated the global burden of 369 diseases and injuries and 87 risk factors, stratified by age and sex, across 204 countries and territories from 1990 to 2021.

The data used in this study were obtained from the official GBD 2021 data platform.^1^ We accessed the “GBD Results Tool” to retrieve relevant data. Under the “GBD Estimate” section, we selected “risk factor” to identify specific exposures contributing to disease burden. In the “Measure” section, we chose both “Death” and “DALYs” to quantify the overall health impact. For the “Risk” category, we selected “diet high in trans-fatty acid” as the exposure of interest, and in the “Cause” section, we selected “cardiovascular disease” as the specific health outcome. In the “Age” section, we selected “55+” and additional age group categories above 55 years to focus on older populations.

By clicking the “Search” button, we were able to filter and extract the raw data relevant to our study; alternatively, the data could be downloaded using the “Download” option. This systematic data extraction process ensured that we obtained accurate and detailed data on the impact of a diet high in trans-fatty acids on cardiovascular disease outcomes, as measured by deaths and DALYs, thereby providing a solid foundation for subsequent burden of disease analysis.

### Burden estimation

The primary objective of this study was to analyze the temporal trends in mortality and disability-adjusted life years (DALY) rates of cardiovascular disease (CVD) attributable to diets high in trans-fatty acids from 1990 to 2019. To eliminate the impact of differences in population structure, we used age-standardized mortality rates (ASMR) and age-standardized DALY rates (ASDR) as key indicators. Trends in ASMR and ASDR were evaluated using the estimated annual percentage change (EAPC), a widely used metric for assessing changes in age-standardized rates (ASR). The ASR per 100,000 population was calculated using the following formula:


$$\mathrm{ASR}=\frac{\sum \begin{array}{c} A\\ i=1 \end{array}a_{i}w_{i}}{\sum \begin{array}{c} A\\ i=1 \end{array}w_{i}}\times 100000$$ (1)

In this formula (1), aᵢ denotes the age-specific rate for the *i*th age group, wᵢ is the number of individuals in the corresponding *i*th age group of the standard population, and A is the total number of age groups. To assess temporal trends in incidence, mortality, and DALYs, we calculated the Estimated Annual Percentage Change (EAPC), a common metric in epidemiological research for evaluating changes in age-standardized rates (ASRs). The EAPC is derived from the regression coefficient (*β*), obtained by modeling the natural logarithm of ASR (ln(ASR)) as a function of calendar year (*x*), using the following linear regression model formula (2):


y=α+βx+ε (2)

EAPC=100×(exp(β)−1) (3)

The direction of ASR trends was assessed by examining the Estimated Annual Percentage Change (EAPC) alongside its 95% confidence interval (CI) formula (3). An increasing trend was identified when both the EAPC and the lower bound of its 95% CI were greater than zero, whereas a decreasing trend was indicated when both the EAPC and the upper bound of the 95% CI were less than zero. To forecast the future burden of disease from 1990 to 2035, we applied a log-linear age-period-cohort (APC) model.

### Sex-, age-, and region-specific analysis

We analyzed sex-specific and age-specific burden across SDI quintiles. Age groups were categorized in 5-year intervals (55–59, 60–64, …, 95+). Comparisons were made between males and females and across SDI levels.

### Age-period-cohort analysis

We applied the Age-Period-Cohort (APC) model using the Intrinsic Estimator method to disentangle age, period, and cohort effects on CVD mortality and DALY burden attributable to high TFA intake.

- Age effect reflects the variation in risk associated with biological and behavioral factors at different ages.
- Period effect represents temporal influences such as medical advancements, public health interventions, or policy changes (e.g., TFA bans).
- Cohort effect captures generational differences in exposure and risk accumulation.
  We also conducted local drift analysis to estimate the annual percentage change in mortality and DALY rates across age groups.

We fitted a log-linear Poisson APC model with polynomial parameterization (quadratic age, period, and cohort effects plus higher-order categorical deviations). The model addresses identifiability through mean-centering, where linear age trend (LAT), net drift, and cohort age trend (CAT) satisfy LAT = Net Drift + CAT. We estimated net drift (overall annual percentage change), local drifts (age-specific trends), and age-period-cohort deviations. Statistical significance was assessed using Wald tests. Analysis used R 4.5.1 with custom functions following Holford (17) and Clayton and Schifflers (18).

### Socioeconomic inequality assessment

We quantified cross-country health inequalities using the Slope Index of Inequality (SII) and Concentration Index (CI).

- SII measures absolute inequality by regressing health outcomes on the relative socioeconomic rank of countries.
- CI assesses relative inequality, with positive values indicating concentration in higher-SDI countries and negative values indicating concentration in lower-SDI countries.

### Bayesian age-period-cohort projection

To predict future trends, we employed a Bayesian Age-Period-Cohort (BAPC) model, incorporating historical ASMR and ASDR data from 1990–2021 to project CVD burden trends up to 2035. The BAPC analysis was implemented using the BAPC R package, which employs Bayesian Age-Period-Cohort modeling via Integrated Nested Laplace Approximation (INLA) (19).

### Model specification

- Latent random effects: Age, period, and cohort effects were defined as latent Gaussian random effects, each specified with first-order random walk (RW1) priors to enable flexible smoothing of temporal patterns. The model was fitted using the BAPC: BAPC() function with secondDiff = FALSE, confirming the use of RW1 smoothing for temporal effects.
- Data structure: The analysis included 20 age groups (stratified in 5-year intervals from 55–59 to 95+) and 32 periods (1990–2021), with cohorts implicitly derived from the Lexis diagram (cohort = period − age).
- Age-standardization: WHO world standard population weights (2000–2025) were applied to ensure consistency with the age-standardization approach used for burden estimation.
- Forecasting settings: Future projections were conducted with predict = list(npredict = 15, retro = TRUE) (to project 15 years beyond the historical data) and a generation factor of gf = 5, aligning with standard cohort analysis conventions.
- Parameterization and Prior Distributions:
- Parameterization difference: Unlike traditional polynomial APC models— which rely on quadratic age, period, and cohort terms with mean-centering constraints—the BAPC approach uses Bayesian random walk priors. This design allows for more flexible capture of non-linear temporal trends and explicitly incorporates uncertainty through posterior distributions, which is critical for interpreting projection reliability.
- Prior specifications: Precision parameters for latent effects adopted default INLA log-gamma priors, while temporal effects (age, period, cohort) were assigned RW1 priors to balance smoothness and responsiveness to trend changes. The choice of priors was guided by previous literature on BAPC modeling for disease burden projections and validated through sensitivity analyses to ensure robustness.

### Statistical analysis

All analyses were conducted using R software (version 4.2.2) with packages Epi, BAPC, and ggplot2. A *p*-value <0.05 was considered statistically significant.

## Results

### Trends in CVD burden attributable to diet high in TFA

Between 1990 and 2021, the number of CVD deaths attributable to high TFA intake among individuals aged 55 and older decreased from 104 million in 1990 to 71.32 million in 2021, marking a 32% reduction (95% CI: −0.33 to −0.31). The ASMR declined from 15.5 per 100,000 to 4.8 per 100,000, representing a 69% decrease (EAPC: -3.7, 95% CI: −4.1 to −3.3), reflecting the positive impact of dietary changes, policy interventions (such as TFA bans), and medical improvements.

**Figure 1:**
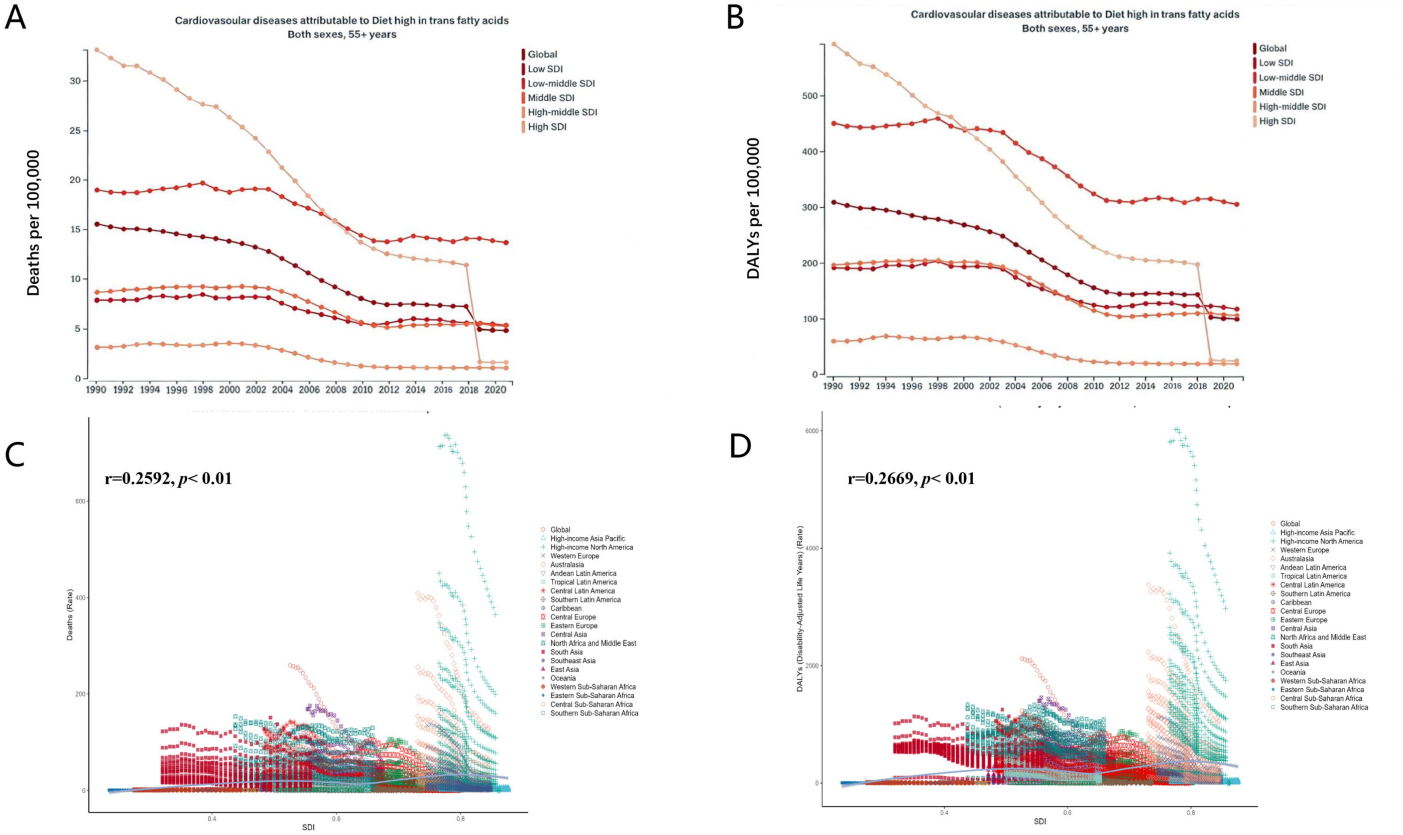
Trends in cardiovascular disease burden attributable to a diet high in trans-fatty acids among adults aged 55 and older from 1990 to 2019. **(A)** Trends in mortality trends. **(B)** Trends in DALY rates. **(C)** The correlation between ASMR and the SDI; **(D)** The correlation between ASDR and the SDI.

The most notable improvements were observed in high-SDI regions, where the number of deaths decreased by 91%, the ASMR dropped from 33.10 to 1.60, and the EAPC was −7.1%. In contrast, low-SDI regions saw an increase in the number of deaths, although mortality rates showed a slight decline (EAPC: −1.6%). Geographically, the fastest declines in mortality rates were recorded in high-income North America (EAPC: −17.3%), Central Europe (−8.4%), and the Caribbean (−7.5%), while more modest declines were seen in North Africa and the Middle East (−0.9%), East Africa (−3.4%), and low-income Asia (−1.5%). Overall, significant global progress has been made in CVD prevention and control; however, regional disparities persist, particularly in areas with limited medical resources, which continue to face considerable challenges (Table 1; Figure 1A).

**Table 1:** Cardiovascular disease mortality burden and age-standardized mortality rate trends attributable to high trans-fatty acid intake among adults aged 55 and older between 1990 and 2021 at the global and regional level.

Location	Deaths 1990 (number) 95%UI	ASR 1990	Deaths 2021 (number) 95%UI	ASR 2021	Number % change	Deaths 1990 (rate)	Deaths 2021 (rate)	EAPC (95% CI)
Global	104399.00 (10561.00–196924.60)	15.5 (1.6, 29.3)	71325.40 (6875.10–139374.90)	4.8 (0.5, 9.4)	−0.32 (−0.97, 12.20)	15.50 (1.60–29.30)	4.80 (0.50–9.40)	−3.7 (−4.1, −3.4)
Low SDI	2944.60 (333.90–5923.60)	7.9 (0.9, 15.9)	4365.50 (406.00–8623.70)	5.3 (0.5, 10.5)	0.48 (−0.93, 24.83)	7.90 (0.90–15.90)	5.30 (0.50–10.50)	−1.6 (−1.9, −1.4)
Low-middle SDI	19163.60 (1932.90–36490.30)	19 (1.9, 36.2)	33014.50 (3019.40–61952.30)	13.7 (1.3, 25.7)	0.72 (−0.92, 31.05)	19.00 (1.90–36.20)	13.70 (1.30–25.70)	-1.4 (−1.6, −1.2)
Middle SDI	15098.10 (1651.20–28764.30)	8.7 (1, 16.6)	24671.20 (2471.70–47512.60)	5.3 (0.5, 10.1)	0.63 (−0.91, 27.77)	8.70 (1.00–16.60)	5.30 (0.50–10.10)	−2.4 (−2.7, −2.0)
High-middle SDI	5399.20 (568.60–11009.00)	3.1 (0.3, 6.4)	3721.40 (359.90–7932.50)	1.1 (0.1, 2.3)	−0.31 (−0.97, 12.95)	3.10 (0.30–6.40)	1.10 (0.10–2.30)	−4.9 (−5.6, −4.2)
High SDI	61734.80 (5888.40–117349.80)	33.1 (3.2, 62.9)	5527.40 (571.60–11261.90)	1.6 (0.2, 3.3)	−0.91 (−1.00, 0.91)	33.10 (3.20–62.90)	1.60 (0.20–3.30)	−7.1 (−8.7, −5.4)
Andean Latin America	374.80 (35.90–729.20)	11.2 (1.1, 21.7)	489.80 (46.10–991.40)	4.9 (0.5, 10)	0.31 (−0.94, 26.62)	11.20 (1.10–21.70)	4.90 (0.50–10.00)	−3.4 (−3.9, −2.8)
Australasia	1586.30 (134.50–3070.00)	40.3 (3.4, 77.9)	632.30 (61.10–1274.10)	7.2 (0.7, 14.4)	−0.60 (−0.98, 8.47)	40.30 (3.40–77.90)	7.20 (0.70–14.40)	−6.5 (−6.9, −6.1)
Caribbean	8.70 (0.90–20.60)	0.2 (0, 0.5)	3.00 (0.20–7.50)	0 (0, 0.1)	−0.66 (−0.99, 7.33)	0.20 (0.00–0.50)	0.00 (0.00–0.10)	−7.5 (−8.3, −6.8)
Central Asia	1280.30 (118.80–2512.40)	16 (1.5, 31.4)	1062.40 (102.00–2118.60)	7.3 (0.7, 14.6)	−0.17 (−0.96, 16.83)	16.00 (1.50–31.40)	7.30 (0.70–14.60)	−3.2 (−3.9, −2.6)
Central Europe	2583.50 (290.80–5238.60)	9.7 (1.1, 19.8)	433.50 (48.00–922.50)	1.2 (0.1, 2.5)	−0.83 (−0.99, 2.17)	9.70 (1.10–19.80)	1.20 (0.10–2.50)	−8.4 (−9.2, −7.6)
Central Latin America	2006.90 (212.30–3932.60)	14.8 (1.6, 29)	2990.80 (305.30–6003.20)	7 (0.7, 14)	0.49 (−0.92, 27.28)	14.80 (1.60–29.00)	7.00 (0.70–14.00)	−3.5 (−4.0, −3.0)
Central Sub-Saharan Africa	8.20 (0.80–20.00)	0.2 (0, 0.5)	7.80 (0.70–18.80)	0.1 (0, 0.2)	−0.05 (−0.97, 22.50)	0.20 (0.00–0.50)	0.10 (0.00–0.20)	−4.1 (−4.7, −3.5)
East Asia	2730.90 (276.00–5806.90)	1.8 (0.2, 3.9)	4802.40 (494.10–11025.40)	1.2 (0.1, 2.8)	0.76 (−0.91, 38.95)	1.80 (0.20–3.90)	1.20 (0.10–2.80)	−2.0 (−2.5, −1.5)
Eastern Europe	2843.90 (269.30–5993.10)	5.8 (0.6, 12.3)	461.10 (45.50–1093.90)	0.7 (0.1, 1.8)	−0.84 (−0.99, 3.06)	5.80 (0.60–12.30)	0.70 (0.10–1.80)	−6.7 (−7.7, −5.7)
Eastern Sub-Saharan Africa	17.00 (1.90–38.10)	0.1 (0, 0.3)	18.50 (1.80–42.60)	0.1 (0, 0.2)	0.09 (−0.95, 21.42)	0.10 (0.00–0.30)	0.10 (0.00–0.20)	−3.4 (−3.9, −2.8)
High-income Asia Pacific	500.70 (45.20–990.90)	1.4 (0.1, 2.8)	803.90 (69.20–1652.00)	1.1 (0.1, 2.3)	0.61 (−0.93, 35.55)	1.40 (0.10–2.80)	1.10 (0.10–2.30)	−1.5 (−1.8, −1.2)
High-income North America	43985.70 (4052.60–83319.30)	75.9 (7, 143.8)	2.30 (0.20–4.30)	0 (0, 0)	−1.00 (−1.00, −1.00)	75.90 (7.00–143.80)	0.00 (0.00–0.00)	−17.3 (−24.6, −9.4)
North Africa and Middle East	8395.30 (799.30–15679.10)	29.7 (2.8, 55.5)	16563.10 (1392.00–32198.20)	21.7 (1.8, 42.2)	0.97 (−0.91, 39.28)	29.70 (2.80–55.50)	21.70 (1.80–42.20)	−0.9 (−1.2, −0.6)
Oceania	2.20 (0.20–5.30)	0.5 (0, 1.1)	2.20 (0.20–5.40)	0.2 (0, 0.4)	0.00 (−0.96, 26.00)	0.50 (0.00–1.10)	0.20 (0.00–0.40)	−4.3 (−4.9, −3.7)
South Asia	22806.60 (2448.20–44358.40)	24 (2.6, 46.7)	38006.90 (3557.00–74448.10)	15.3 (1.4, 30)	0.67 (−0.92, 29.41)	24.00 (2.60–46.70)	15.30 (1.40–30.00)	−2.1 (−2.5, −1.8)
Southeast Asia	126.80 (13.40–266.60)	0.3 (0, 0.6)	137.20 (13.30–310.60)	0.1 (0, 0.3)	0.08 (−0.95, 22.18)	0.30 (0.00–0.60)	0.10 (0.00–0.30)	−4.1 (−4.7, −3.6)
Southern Latin America	88.70 (9.00–188.40)	1.1 (0.1, 2.4)	31.60 (3.10–72.20)	0.2 (0, 0.5)	−0.64 (−0.98, 7.02)	1.10 (0.10–2.40)	0.20 (0.00–0.50)	−5.8 (−6.2, −5.4)
Southern Sub-Saharan Africa	1.00 (0.10–2.90)	0 (0, 0.1)	0.10 (0.00–0.30)	0 (0, 0)	−0.90 (−1.00, 2.00)	0.00 (0.00–0.10)	0.00 (0.00–0.00)	−12.2 (−14.4, −10.0)
Tropical Latin America	1423.90 (161.60–2909.60)	9.4 (1.1, 19.2)	1145.10 (109.00–2300.90)	2.6 (0.2, 5.2)	−0.20 (−0.96, 13.24)	9.40 (1.10–19.20)	2.60 (0.20–5.20)	−4.9 (−5.3, −4.5)
Western Europe	13593.90 (1358.10–26756.20)	14 (1.4, 27.6)	3698.10 (398.00–7579.40)	2.5 (0.3, 5.1)	−0.73 (−0.99, 4.58)	14.00 (1.40–27.60)	2.50 (0.30–5.10)	−6.6 (−7.1, −6.1)
Western Sub-Saharan Africa	33.50 (2.80–76.20)	0.2 (0, 0.5)	33.40 (3.70–74.40)	0.1 (0, 0.2)	−0.00 (−0.95, 25.57)	0.20 (0.00–0.50)	0.10 (0.00–0.20)	−3.4 (−4.0, −2.8)

**Figure 2:**
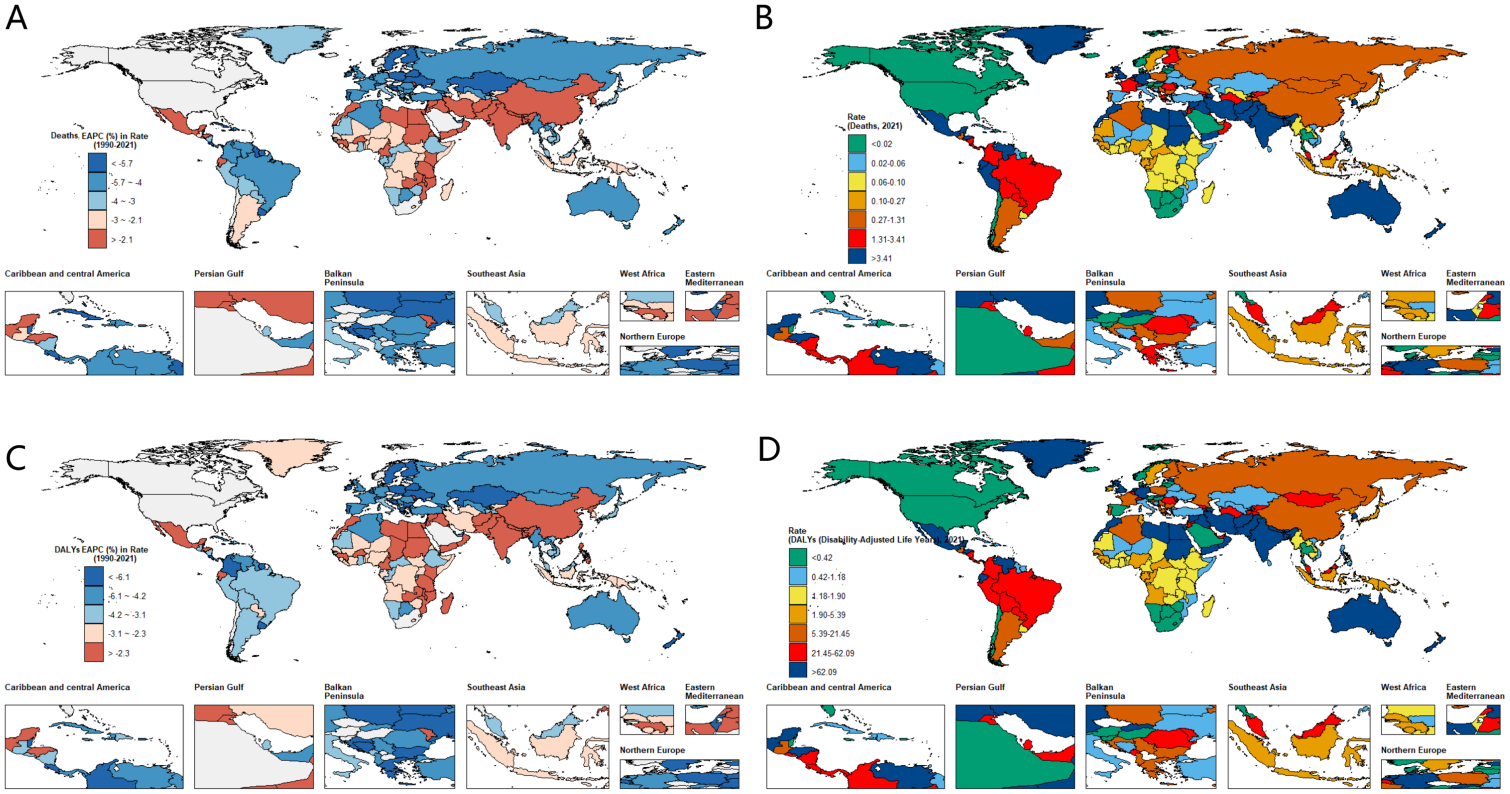
Global burden of cardiovascular disease attributable to a diet high in trans-fatty acids among adults aged 55 and older from 1990 to 2019 across 204 countries and territories. **(A)** EAPC in ASMR from CVD. **(B)** ASMR of CVD. **(C)** EAPC in ASDR from CVD. **(D)** ASDR of CVD.

At the national level, the number of deaths in China increased by 76.3%, and in India by 64.3%; however, their ASMR declined from 1.9 to 1.3 and from 24.7 to 15.5, respectively. In contrast, Germany saw a 71.5% decrease in deaths, and the UK a 75.4% decrease, both accompanied by substantial reductions in ASMR, reflecting effective disease prevention and treatment strategies. Regionally, South Asia—particularly Pakistan (ASMR: 18.5)—bears a heavy burden of CVD; similarly, Egypt (ASMR: 95.9) and Iran (ASMR: 36.1) in North Africa and the Middle East also report high levels. The most significant improvements were seen in Eastern Europe’s Georgia, where ASMR dropped from 25.2 to 1.8; Western Europe’s Ireland, with an EAPC of −11.1%; and Central Asia’s Kazakhstan, with an EAPC as high as −14.4% (Supplementary Table S1; Figure 2).

In terms of DALYs, from 1990 to 2021, the global burden of CVD attributable to TFA intake showed a declining trend both globally and across different SDI regions. The global ASDR dropped from 309.6 per 100,000 in 1990 (95% UI: 31.1–581.3) to 100.1 in 2021 (95% UI: 9.4–193.2). In low SDI regions, the ASDR decreased from 192.1 (95% UI: 22.0–384.5) to 118.2 (95% UI: 11.2–235.1); in low-middle SDI regions, from 450.9 (95% UI: 45.9–856.9) to 306.4 (95% UI: 28.3–578.0); in middle SDI regions, from 196.9 (95% UI: 21.4–378.4) to 106.8 (95% UI: 10.2–208.8); and in high-middle SDI regions, from 61.0 (95% UI: 6.4–124.6) to 18.8 (95% UI: 1.8–38.7). These figures indicate a general reduction in the burden of CVD due to high TFA intake across all regions, with the most significant decline observed in high-middle SDI areas (Table 2; Figure 1B).

**Table 2:** Cardiovascular disease mortality burden and age-standardized DALYs rate trends attributable to high trans-fatty acid intake among adults aged 55 and older between 1990 and 2021 at the global and regional level.

Location	DALYs (disability-adjusted life years) 1990 (number) 95%UI	ASR 1990	DALYs (disability-adjusted life years) 2021 (number) 95%UI	ASR 2021	Number % change	DALYs (disability-adjusted life years) 1990 (Rate)	DALYs (disability-adjusted life years) 2021 (Rate)	EAPC (95% CI)
Global	2078913.20 (208671.30–3903147.20)	309.6 (31.1, 581.3)	1487292.90 (140292.20–2870862.00)	100.1 (9.4, 193.2)	−0.28 (−0.96, 12.76)	309.60 (31.10–581.30)	100.10 (9.40–193.20)	−3.7 (−4.0, −3.3)
Low SDI	71680.70 (8193.80–143456.90)	192.1 (22, 384.5)	96958.60 (9217.90–192935.00)	118.2 (11.2, 235.1)	0.35 (−0.94, 22.55)	192.10 (22.00–384.50)	118.20 (11.20–235.10)	−2.1 (−2.4, −1.7)
Low-middle SDI	454525.10 (46282.40–863745.10)	450.9 (45.9, 856.9)	738713.70 (68342.10–1393452.50)	306.4 (28.3, 578)	0.63 (−0.92, 29.11)	450.90 (45.90–856.90)	306.40 (28.30–578.00)	−1.6 (−1.8, −1.4)
Middle SDI	341721.80 (37057.90–656781.80)	196.9 (21.4, 378.4)	501767.40 (47728.30–980876.90)	106.8 (10.2, 208.8)	0.47 (−0.93, 25.47)	196.90 (21.40–378.40)	106.80 (10.20–208.80)	−2.8 (−3.2, −2.4)
High-middle SDI	105270.60 (11085.00–214957.30)	61 (6.4, 124.6)	65159.40 (6296.00–134210.00)	18.8 (1.8, 38.7)	−0.38 (−0.97, 11.11)	61.00 (6.40–124.60)	18.80 (1.80–38.70)	−5.3 (−6.0, −4.6)
High SDI	1104602.20 (105763.30–2085564.90)	592.4 (56.7, 1118.5)	84201.00 (8402.80–168882.00)	24.4 (2.4, 48.9)	−0.92 (−1.00, 0.60)	592.40 (56.70–1118.50)	24.40 (2.40–48.90)	−7.3 (−9.0, −5.6)
Andean Latin America	7156.70 (667.90–14049.80)	213.3 (19.9, 418.7)	8846.40 (850.70–17856.50)	89.3 (8.6, 180.3)	0.24 (−0.94, 25.74)	213.30 (19.90–418.70)	89.30 (8.60–180.30)	−3.7 (−4.2, −3.1)
Australasia	29161.50 (2448.90–55589.10)	740.2 (62.2, 1411.1)	9498.80 (856.20–18716.80)	107.5 (9.7, 211.9)	−0.67 (−0.98, 6.64)	740.20 (62.20–1411.10)	107.50 (9.70–211.90)	−7.2 (−7.6, −6.8)
Caribbean	158.00 (16.30–368.10)	3.7 (0.4, 8.5)	52.70 (3.20–142.40)	0.6 (0, 1.5)	−0.67 (−0.99, 7.74)	3.70 (0.40–8.50)	0.60 (0.00–1.50)	−7.6 (−8.4, −6.8)
Central Asia	26214.50 (2513.40–50845.90)	327.8 (31.4, 635.7)	21498.50 (2142.10–42514.30)	147.8 (14.7, 292.2)	−0.18 (−0.96, 15.92)	327.80 (31.40–635.70)	147.80 (14.70–292.20)	−3.6 (−4.2, −2.9)
Central Europe	49030.90 (5427.20–98952.50)	184.9 (20.5, 373.1)	6815.30 (752.70–14226.50)	18.4 (2, 38.4)	−0.86 (−0.99, 1.62)	184.90 (20.50–373.10)	18.40 (2.00–38.40)	−9.1 (−9.9, −8.2)
Central Latin America	39412.00 (4200.70–77387.70)	290.4 (31, 570.3)	54506.40 (5643.20–110147.60)	127.5 (13.2, 257.6)	0.38 (−0.93, 25.22)	290.40 (31.00–570.30)	127.50 (13.20–257.60)	−3.9 (−4.4, −3.3)
Central Sub-Saharan Africa	187.40 (18.90–470.40)	5 (0.5, 12.5)	170.90 (14.80–420.70)	1.9 (0.2, 4.7)	−0.09 (−0.97, 21.26)	5.00 (0.50–12.50)	1.90 (0.20–4.70)	−4.2 (−4.8, −3.7)
East Asia	57438.30 (6030.30–121906.00)	38.6 (4, 81.8)	79955.20 (8028.00–182208.50)	20.4 (2, 46.5)	0.39 (−0.93, 29.22)	38.60 (4.00–81.80)	20.40 (2.00–46.50)	−2.8 (−3.3, −2.3)
Eastern Europe	52608.20 (5046.00–110940.10)	107.6 (10.3, 226.9)	7855.00 (805.60–18226.30)	12.7 (1.3, 29.4)	−0.85 (−0.99, 2.61)	107.60 (10.30–226.90)	12.70 (1.30–29.40)	−7.3 (−8.3, −6.2)
Eastern Sub-Saharan Africa	388.90 (39.80–893.60)	3.2 (0.3, 7.3)	393.80 (38.40–887.20)	1.5 (0.1, 3.3)	0.01 (−0.96, 21.29)	3.20 (0.30–7.30)	1.50 (0.10–3.30)	−3.6 (−4.2, −3.1)
High-income Asia Pacific	9795.80 (822.20–19071.20)	28 (2.4, 54.5)	12725.10 (1037.20–25437.80)	18 (1.5, 36.1)	0.30 (−0.95, 29.94)	28.00 (2.40–54.50)	18.00 (1.50–36.10)	−2.2 (−2.6, −1.9)
High-income North America	791144.00 (74416.70–1480967.50)	1365.7 (128.5, 2556.6)	52.50 (4.30–102.80)	0 (0, 0.1)	−1.00 (−1.00, −1.00)	1365.70 (128.50–2556.60)	0.00 (0.00–0.10)	−17.0 (−24.0, −9.2)
North Africa and Middle East	189985.30 (17697.70–353377.90)	672.2 (62.6, 1250.3)	360425.40 (30858.60–697382.50)	472.8 (40.5, 914.8)	0.90 (−0.91, 38.41)	672.20 (62.60–1250.30)	472.80 (40.50–914.80)	−1.1 (−1.3, −0.8)
Oceania	52.80 (4.70–129.90)	11 (1, 27)	49.40 (4.30–128.20)	4 (0.4, 10.4)	−0.06 (−0.97, 26.28)	11.00 (1.00–27.00)	4.00 (0.40–10.40)	−4.4 (−4.9, −3.8)
South Asia	554919.40 (59743.70–1073878.80)	584.5 (62.9, 1131.1)	840636.50 (77101.70–1703698.70)	338.6 (31.1, 686.2)	0.51 (−0.93, 27.52)	584.50 (62.90–1131.10)	338.60 (31.10–686.20)	−2.5 (−2.9, −2.1)
Southeast Asia	2716.80 (280.10–5958.60)	6.4 (0.7, 14.1)	2860.50 (297.40–6438.30)	2.5 (0.3, 5.6)	0.05 (−0.95, 21.99)	6.40 (0.70–14.10)	2.50 (0.30–5.60)	−4.2 (−4.8, −3.7)
Southern Latin America	1610.60 (158.80–3462.20)	20.3 (2, 43.7)	534.70 (48.60–1244.10)	3.6 (0.3, 8.5)	−0.67 (−0.99, 6.83)	20.30 (2.00–43.70)	3.60 (0.30–8.50)	−6.1 (−6.5, −5.7)
Southern Sub-Saharan Africa	19.20 (1.30–50.40)	0.4 (0, 1.1)	2.30 (0.00–7.10)	0 (0, 0.1)	−0.88 (−1.00, 4.46)	0.40 (0.00–1.10)	0.00 (0.00–0.10)	−12.0 (−14.1, −9.9)
Tropical Latin America	30734.00 (3425.50–64056.20)	203 (22.6, 423.1)	24034.80 (2259.20–50070.20)	54.3 (5.1, 113)	−0.22 (−0.96, 13.62)	203.00 (22.60–423.10)	54.30 (5.10–113.00)	−5.1 (−5.5, −4.6)
Western Europe	235499.40 (23837.90–454769.10)	242.5 (24.5, 468.3)	55718.00 (5704.20–112348.20)	37.4 (3.8, 75.3)	−0.76 (−0.99, 3.71)	242.50 (24.50–468.30)	37.40 (3.80–75.30)	−7.0 (−7.5, −6.5)
Western Sub-Saharan Africa	679.60 (58.10–1499.10)	4.7 (0.4, 10.4)	660.60 (69.30–1450.10)	2.1 (0.2, 4.5)	−0.03 (−0.95, 23.96)	4.70 (0.40–10.40)	2.10 (0.20–4.50)	−3.5 (−4.1, −2.9)

At the national level, taking China as an example, the total DALYs increased from 57,176.1 to 79,733.1, representing a 39.5% rise. However, ASDR declined from 39.8 to 21.0, marking a 47.2% decrease. Globally, EAPC in ASDR was −4.6%. Kazakhstan showed the best performance, with an EAPC of −14.9%, while Georgia’s ASDR dropped significantly from 478.6 to 31.2, with an EAPC of −11.5%. The countries with the heaviest burden in 2021 included Egypt (ASDR as high as 2208.4), India (342.9), and Pakistan (417.8). Notably, Libya exhibited an abnormal upward trend, with its ASDR increasing from 52.1 to 76.5 and an EAPC of +2.1% (Supplementary Table S2; Figure 2).

Figures 1C,D show a significant positive correlation between ASMR and ASDR and the SDI (*p* < 0.01). Although the data indicate that higher SDI levels are associated with higher ASMR and ASDR values, overall, both ASMR and ASDR of CVD attributable to high TFA intake have declined globally and across all SDI regions. Moreover, the degree of reduction in disease burden is positively associated with SDI level, suggesting that higher-income countries have achieved more notable progress through effective interventions and improvements.

### CVD burden attributable to high trans-fatty acid intake by sex across SDI regions in adults aged 55+

**Figure 3:**
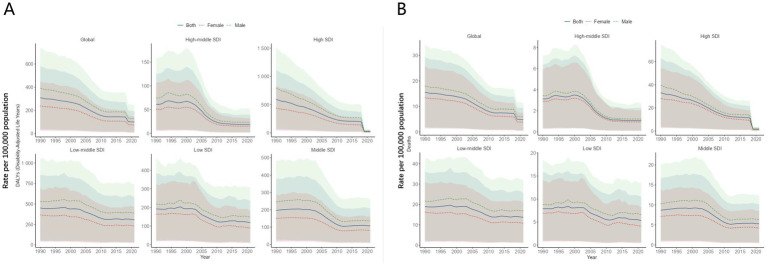
CVD burden attributable to high TFA intake by sex across SDI regions in adults aged 55+. **(A)** DALYs rate of CVD attributable to high TFA intake by sex across SDI regions. **(B)** Mortality of CVD attributable to high TFA intake by sex across SDI regions.

From 1990 to 2021, the burden of CVD attributable to high TFA intake among adults aged 55 and older exhibited notable sex differences across different SDI regions. Overall, males consistently bore a higher burden than females in nearly all SDI levels, in terms of both DALYs and mortality. This disparity was most pronounced in low and low-middle SDI regions, where the gaps between sexes were particularly large. In contrast, high SDI regions showed relatively minor differences between males and females, with their DALYs and mortality rates converging, reflecting more equitable health interventions and access to resources. Thus, while sex disparities in CVD burden are globally prevalent, they are especially significant in regions with lower SDI levels (Figures 3A,B).

### Sex- and age-specific CVD mortality attributable to high TFA intake in adults aged 55 + worldwide

**Figure 4:**
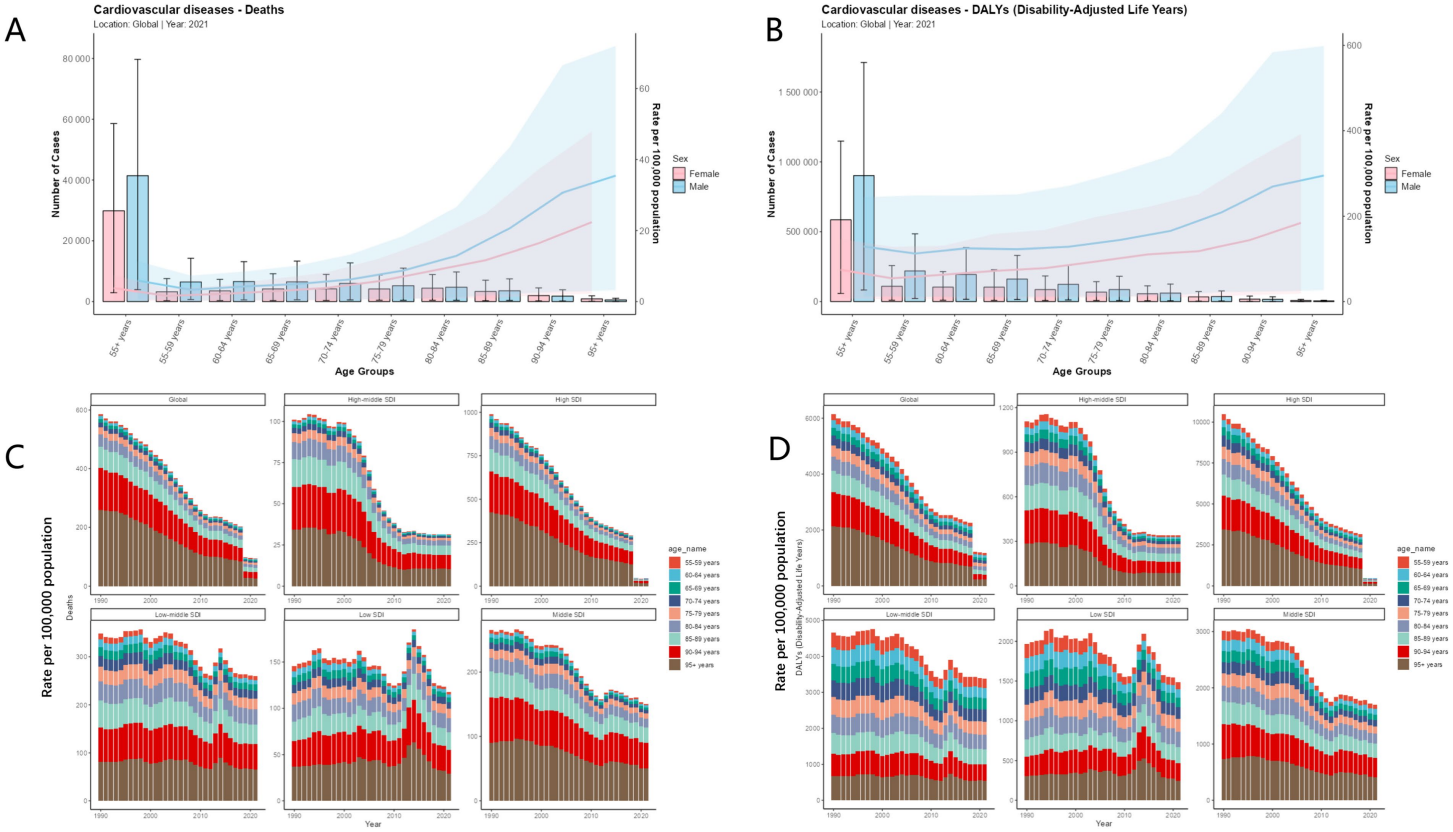
Sex- and age-specific CVD mortality attributable to high TFA intake in adults aged 55+ worldwide. **(A)** Age-specific male-to-female ratios of CVD mortality attributable to high TFA intake in 2021. **(B)** Age-specific male-to-female ratios of CVD DALYs rate attributable to high TFA intake in 2021. **(C)** Age-specific proportions of CVD mortality attributable to high TFA intake (1990–2021). **(D)** Age-specific proportions of CVD DALYs rate attributable to high TFA intake (1990–2021).

We conducted a global analysis of sex- and age-specific differences in CVD attributable to high TFA intake among adults aged 55 and older. As shown in Figures 4A,B, in terms of sex differences, the CVD mortality and DALYs rate attributable to high TFA intake in 2021 was significantly higher in males than in females across the 55 + age group, reflecting the global epidemiological pattern of CVD. Regarding age differences, we found that the absolute numbers of deaths and DALYs were highest in the 55–59, 60–64, and 65–69 age groups. However, both mortality rate and DALY rate increased markedly with age, with a sharp rise observed among individuals aged 80 and above. The highest rates were recorded in the 95 + age group, while the lowest were in the 55–59 age group. As shown in Figures 4C,D, the burden of deaths and DALYs across different age groups also varies by SDI level. In high SDI regions, due to a higher degree of population aging, the disease burden is more heavily concentrated in the 85 + age group. In contrast, in low SDI regions, the burden is more prominent in younger age groups, particularly among those aged 55–64. Over time, from 1990 to 2021, the age distribution of CVD burden has shifted. The proportion of the burden among the middle-aged and elderly groups (55–79 years) has steadily increased, while the proportion in the 95 + age group has declined. This indicates a gradual shift in CVD burden from the oldest old to the younger elderly population. Additionally, the burden proportions for the 75–79 and 80–84 age groups have also increased, highlighting that middle-aged age groups are becoming the new focus of disease burden. In contrast, the decreasing share among those aged 90 and above reflects an ongoing and structured transformation in the age distribution of CVD burden attributable to high TFA intake, driven by changes in population structure and healthcare conditions worldwide.

### Aged-period-cohort analysis for CVD attributable to high trans-fatty acid intake in adults aged 55 + worldwide

**Figure 5:**
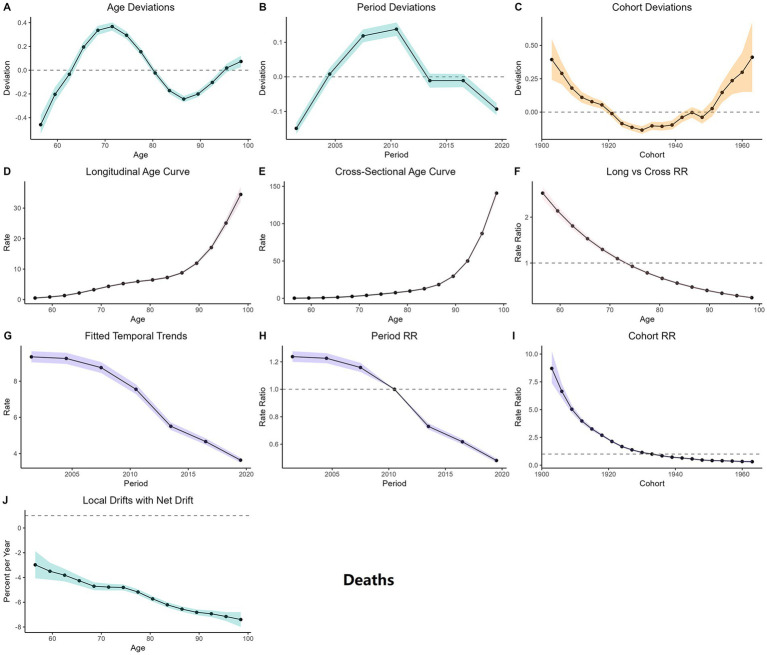
APC analysis of CVD mortality attributable to high TFA intake from 1990 to 2021. **(A)** Age deviations: solid line represents the deviation of mortality risk from the overall mean across age groups (55–59 to 95+); positive values indicate higher-than-average risk, negative values indicate lower-than-average risk. **(B)** Period deviation: solid line denotes the deviation of mortality risk from the overall mean across periods (1990–2021); fluctuations reflect temporal shifts (e.g., policy interventions, medical advancements). **(C)** Cohort deviation: solid line shows the deviation of mortality risk from the overall mean across birth cohorts (1900–1985); differences reflect generational exposure to TFA and other risk factors. **(D)** Longitudinal age curve: solid line represents the age-specific mortality risk trajectory for a fixed cohort as it ages; estimated from cohort effects. **(E)** Cross-section age curve: solid line denotes the age-specific mortality risk observed at a single time point (pooled across periods); observed data aggregated by age. **(F)** Long vs. cross RR: bars compare the relative risk (RR) of mortality from longitudinal (fixed cohort) vs. cross-sectional (time-point aggregated) analyses; estimated RR values. **(G)** Fitted temporal trend: solid line shows the model-fitted trend of age-standardized mortality rates (ASMR) from 1990 to 2021; estimated to align with observed data. **(H)** Period RR: solid line represents the relative risk of mortality across periods (1990–2021) compared to the reference period; estimated via APC modeling. **(I)** Cohort RR: solid line denotes the relative risk of mortality across birth cohorts compared to the reference cohort; estimated via APC modeling. **(J)** Local drifts with net drift: solid line shows the net annual percentage change in mortality risk across age groups; shaded areas represent 95% confidence intervals (CIs) for uncertainty; estimated from drift analysis.

**Figure 6:**
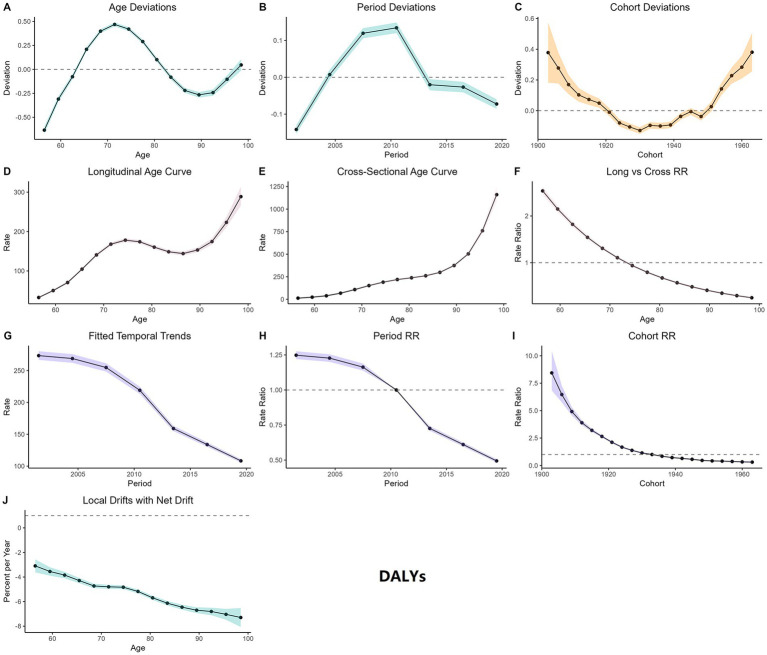
APC analysis of CVD DALYs attributable to high TFA intake from 1990 to 2021. **(A)** Age deviations; solid line represents the deviation of DALY risk from the overall mean across age groups (55–59 to 95+); positive values = higher-than-average risk, negative values = lower-than-average risk. **(B)** Period deviation; solid line denotes the deviation of DALY risk from the overall mean across periods (1990–2021); reflects temporal changes (e.g., dietary interventions). **(C)** Cohort deviation; solid line shows the deviation of DALY risk from the overall mean across birth cohorts (1900–1985); reflects generational risk accumulation. **(D)** Longitudinal age curve; solid line represents the age-specific DALY risk trajectory for a fixed cohort as it ages; estimated from cohort effects. **(E)** Cross-section age curve; solid line denotes the age-specific DALY risk observed at a single time point (pooled across periods); observed data aggregated by age. **(F)** Long vs. cross RR; bars compare the relative risk (RR) of DALYs from longitudinal vs. cross-sectional analyses; estimated RR values. **(G)** Fitted temporal trend; solid line shows the model-fitted trend of age-standardized DALY rates (ASDR) from 1990–2021; estimated to align with observed data. **(H)** Period RR; solid line represents the relative risk of DALYs across periods compared to the reference period; estimated via APC modeling. **(I)** Cohort RR; solid line denotes the relative risk of DALYs across birth cohorts compared to the reference cohort; estimated via APC modeling. **(J)** Local drifts with net drift. Solid line shows the net annual percentage change in DALY risk across age groups; shaded areas = 95% CIs for uncertainty; estimated from drift analysis.

Figures 5, 6 present the results of the Age-Period-Cohort (APC) analysis, indicating that both the mortality and DALY burden of CVD attributable to high TFA intake in adults aged 55 and older increase significantly with age, peaking in individuals aged 80 and above. The age deviation curve shows the greatest risk deviation in the 80–85 age group, followed by slight fluctuations. Period deviations reveal that the risk was slightly higher than the baseline during 2005–2010, followed by a declining trend after 2010. Cohort deviations indicate the highest risk deviations in birth cohorts from 1900 to 1920, the lowest in those born between 1930 and 1945, and a renewed increase in cohorts born after 1950. The cohort relative risk (RR) decreased gradually from the 1900 cohort to the 1940 cohort (with mortality risks 3 to 7 times higher than baseline), then slightly rebounded in cohorts born after 1950. The period RR peaked in 2005 and then declined, falling below the baseline level by 2020. Local drift analysis suggests that the annual rate of risk decline slows with increasing age, with more pronounced reductions observed in the younger elderly groups.

### Cross-country inequality analysis

**Figure 7:**
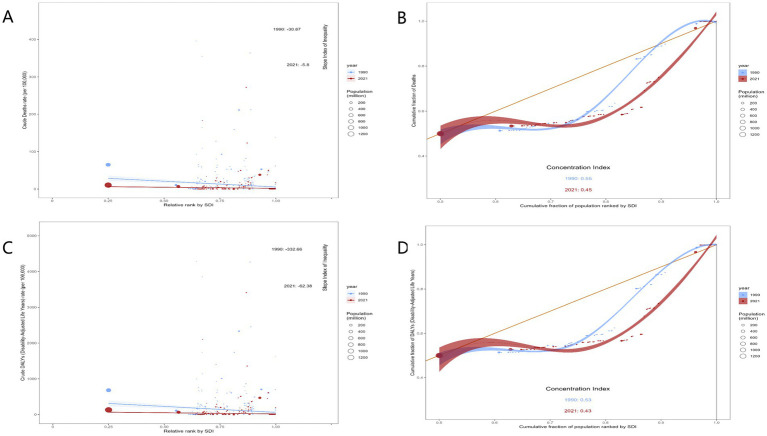
Income-related health inequality regression and concentration curves for the burden of CVD attributable to high TFA intake in adults aged 55+ worldwide, 1990 and 2021. **(A)** Slope index of inequality (SII) for ASMR: Solid lines = regression lines of ASMR against the cumulative fraction of the population ranked by SDI (socioeconomic status); slope = SII (absolute inequality). Dots = observed ASMR values for each country/territory, colored by SDI quintile (high SDI = dark, low SDI = light) to distinguish socioeconomic groups. Values in the panel (1990: −30.87; 2021: −5.8) = estimated SII coefficients, reflecting reduced absolute inequality over time. **(B)** Concentration index (CI) for ASMR: Solid curve = concentration curve; dashed line = line of perfect equality (no socioeconomic inequality). Area between the curve and dashed line = estimated CI (relative inequality); positive CI values (1990: 0.55; 2021: 0.45) indicate burden concentration in higher-SDI populations. Dots = observed ASMR values for each country/territory, colored by SDI quintile. **(C)** Slope index of inequality (SII) for ASDR: Solid lines = regression lines of ASDR against cumulative population ranked by SDI; slope = SII. Dots = observed ASDR values for each country/territory, colored by SDI quintile. Values (1990: −332.88; 2021: −62.38) = estimated SII coefficients, indicating reduced absolute inequality in DALYs. **(D)** Concentration index (CI) for ASDR: Solid curve = concentration curve; dashed line = line of perfect equality. Area between the curve and dashed line = estimated CI; positive values (1990: 0.53; 2021: 0.43) indicate burden concentration in higher-SDI populations. Dots = observed ASDR values for each country/territory, colored by SDI quintile. All regression lines, SII, and CI values are estimated from 1990 and 2021 observed data; no predicted results or bands are included.

As shown in Figure 7, our study revealed significant changes in socioeconomic disparities in CVD mortality and DALYs attributable to high TFA intake among adults aged 55 years and older from 1990 to 2021. In 1990, both CVD mortality and health burden were substantially higher among populations with lower socioeconomic status (SES), with slope index of inequality (SII) values of −30.87 for mortality and −332.66 for DALYs, indicating pronounced health inequality. By 2021, these gaps had narrowed considerably, with SII decreasing to −5.8 for mortality and −62.38 for DALYs, reflecting a notable reduction in absolute health disparities. However, changes in the concentration index (CI) highlighted a clear shift in the pattern of inequality. In 1990, both mortality and DALYs showed a slight concentration among higher SES groups, with CI values of 0.55 and 0.53, respectively. By 2021, these values declined to 0.45 for mortality and 0.43 for DALYs, suggesting that although socioeconomic inequality had improved overall, higher-income populations continued to maintain a significant health advantage.

### Projections for the future

**Figure 8:**
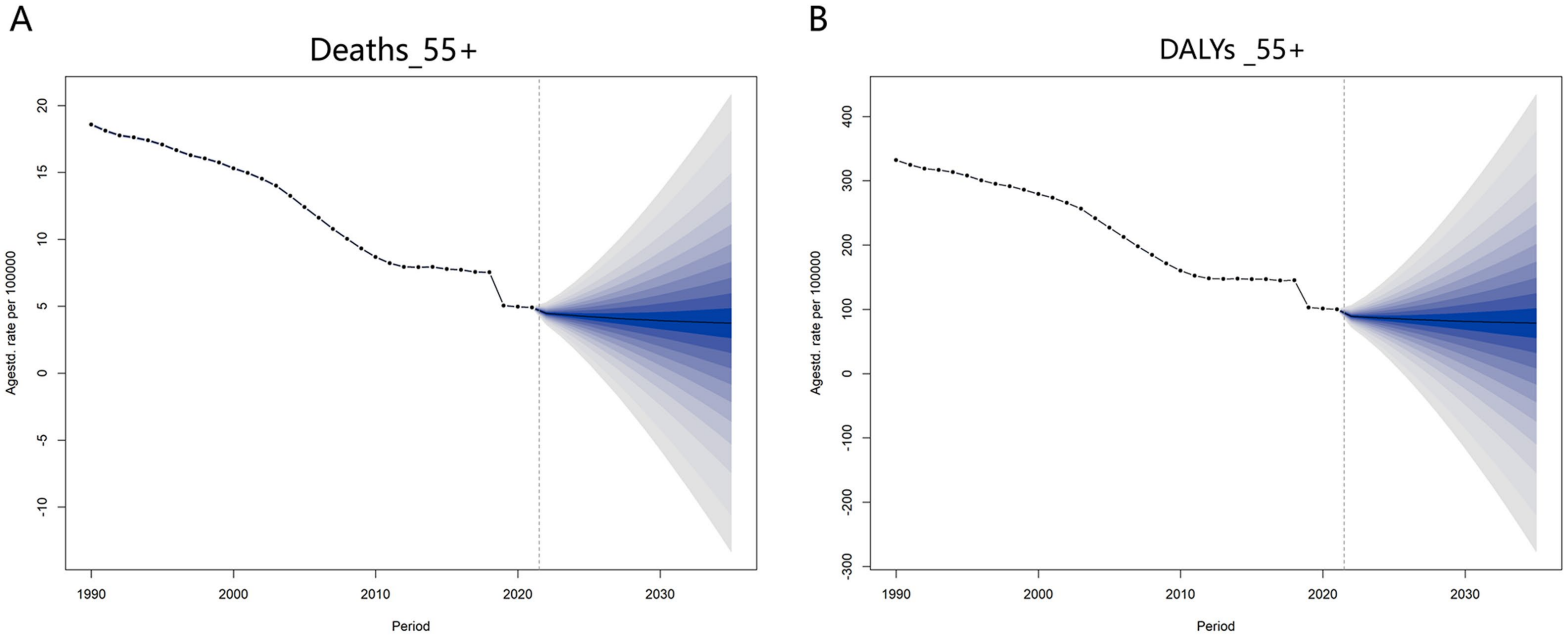
BAPC-projected the global disease burden trends of CVD attributable to high TFA intake among adults aged 55 years and older over the next 15 years (2021–2036). **(A)** Projected ASMR for CVD attributable to high TFA intake in adults aged 55 and older. Solid line (1990–2021) = observed age-standardized mortality rates; solid line (2022–2036) = predicted ASMR from the BAPC model. Shaded band = 95% uncertainty interval (UI) for predicted values, reflecting variability in projections due to demographic, policy, and healthcare factors. **(B)** Projected ASDR for CVD attributable to high TFA intake in adults aged 55 and older. Solid line (1990–2021) = observed age-standardized DALY rates; solid line (2022–2036) = predicted ASDR from the BAPC model. Shaded band = 95% UI for predicted values, quantifying uncertainty in future burden trends. All data for 1990–2021 are observed; 2022–2036 values are predicted via Bayesian age-period-cohort modeling, with shaded bands indicating 95% UI for prediction uncertainty.

Based on ASMR and ASDR data from 1990 to 2021 for individuals aged 55 and above, the BAPC (Bayesian Age-Period-Cohort) model was employed to project the global disease burden trends of CVDs attributable to high TFA intake over the next 15 years (Figure 8). The results indicate a continued decline in both mortality rates and DALYs attributable to high TFA intake. However, the standard deviation (SD) increases significantly over time. By 2035, the global ASMR is projected to further decrease to 3.74 per 100,000, but with a relatively high SD of 8.71. The DALY rate is expected to fall to 78.55 per 100,000. The higher SD suggests that the actual burden may deviate from projections due to potential influences such as accelerated population aging, the rise of emerging diseases, and disparities in access to healthcare resources.

## Discussion

This study provides a comprehensive global assessment of the burden and trends of CVD mortality and DALYs attributable to high TFA intake among adults aged 55 years and older from 1990 to 2021, based on the Global Burden of Disease (GBD) 2021 data. Several important findings have emerged.

First, our analysis shows that the global ASMR and ASDR for CVD attributable to high TFA intake have declined substantially over the past three decades. Between 1990 and 2021, the global ASMR decreased by 69%, while the ASDR fell by approximately 68%. These trends likely reflect the cumulative effects of public health policies restricting TFA use, improvements in dietary patterns, increased awareness of cardiovascular risk factors, and advances in healthcare systems. Notably, high-SDI regions achieved the most substantial reductions in both mortality and DALYs, consistent with previous studies showing that comprehensive policies—including mandatory TFA bans, improved food labeling, and widespread health education—effectively lower population-level TFA exposure and associated cardiovascular risk (16, 20). Fortunately, according to reports by Lindsay Steele, since global efforts accelerated in 2018, 43 countries have adopted best-practice regulations, protecting an additional 3.2 billion people and creating momentum for global TFA elimination (21). By mid-2018, fifteen years after Denmark first pioneered action, 27 countries—mainly high-income nations in Europe and the Americas—had introduced mandatory restrictions on industrial TFA (iTFA), with only 14 implementing regulations as strict as Denmark’s, one of the two best-practice policies recommended by the World Health Organization (WHO). The WHO has emphasized that eliminating iTFA is a highly cost-effective intervention for preventing and controlling non-communicable diseases (NCDs) in low- and middle-income countries. Nevertheless, prior to 2018, few lower-income countries had implemented such restrictions, resulting in excessive iTFA levels in the food supply and contributing to preventable cardiovascular events (22, 23).

Second, our findings reveal pronounced sex and age disparities. Consistent with global CVD epidemiology, men exhibited consistently higher mortality and DALY rates than women. This gap was most pronounced in low SDI countries, likely attributable to more significant gender-based divergences in health behaviors, occupational exposures, and healthcare accessibility. Regarding age distribution, although the highest absolute disease burden occurred among those aged 55–69 years, mortality and DALY rates rose sharply with advancing age, peaking in individuals aged ≥80 years. These trends indicate that the oldest adults are not only more susceptible to dietary risks but also more vulnerable to the cumulative impact of age-related cardiovascular system degeneration (24, 25).

Third, our age-period-cohort analysis offers novel insights into the demographic and temporal drivers of CVD burden related to high TFA intake. Age effects demonstrated an exponential increase in CVD mortality and DALY rates with advancing age, peaking among individuals aged 80 years and older. These results are consistent with the natural history of atherosclerotic cardiovascular disease, where cumulative exposure to risk factors over time leads to increased susceptibility to fatal and disabling events in later life. Furthermore, the shifting age distribution of disease burden toward middle-old groups (55–79 years) suggests an evolving epidemiological profile, with CVD emerging as a significant health challenge for relatively younger elderly populations, especially in low-SDI regions.

Our APC analysis also reveals novel demographic and temporal drivers of CVD burden associated with high TFA intake. Age effects demonstrated an exponential increase in CVD mortality and DALY rates with advancing age, peaking among individuals aged ≥80 years, consistent with the natural progression of atherosclerotic CVD, where cumulative exposure to risk factors elevates susceptibility to fatal and disabling events in later life (26). Notably, the shifting age distribution of disease burden toward middle-aged and older adults (55–79 years) signals an evolving epidemiological profile, with CVD emerging as a critical health challenge for relatively younger elderly populations, particularly in low-SDI regions. Concurrently, cohort effect analysis identified progressively elevated relative risks among post-1950 birth cohorts, suggesting that despite overall preventive advances, newer generations face persistent or emerging exposures to cardiovascular risk factors, potentially linked to dietary transitions, urbanization, and contemporary lifestyle challenges (27).

Our inequality analysis revealed substantial reductions in absolute health disparities over time, with the SII for both mortality and DALYs narrowing significantly between 1990 and 2021. However, concentration index trends demonstrate persistently entrenched socioeconomic inequalities, reflecting a growing concentration of CVD burden among disadvantaged populations. These results further highlight the persistent influence of social factors such as income, education, and access to healthcare on health outcomes (28, 29), reinforcing the need for targeted interventions that promote health equity among disadvantaged populations.

Our projections suggest that the global burden of CVD attributable to TFA intake will continue to decline through 2036. However, the widening predictive intervals indicate increasing uncertainty, primarily driven by factors such as global population aging, the rising prevalence of metabolic diseases, and uneven policy implementation, particularly in low SDI countries. In some high-income countries, legislative measures, food labeling policies, and industry reform have effectively curbed TFA intake. For instance, a study from the UK suggests that a total ban on TFAs in processed foods could prevent or delay approximately 7,200 deaths from coronary heart disease between 2015 and 2020, reduce related health inequalities by 15%, and generate net economic benefits of up to £265 million. By contrast, measures such as improved labeling or restricting TFAs in the food service sector would be less than half as effective (30). Nevertheless, many low- and middle-income countries still face weak regulatory frameworks, resource shortages, and industry interference, meaning health inequalities remain largely unresolved. In fact, in many of these countries, the primary source of TFAs is not pre-packaged food but products sold by street vendors and within the informal food sector (31, 32), which significantly limits the effectiveness of labeling-based interventions. Moreover, the rapid growth of the elderly population in developing regions, combined with the rising burden of metabolic diseases, may undermine existing policy achievements and lead to a rebound in CVD mortality risk among vulnerable groups (33). Therefore, there is an urgent need to strengthen global coordination and governance, promote the complete elimination of industrial TFAs, and integrate dietary interventions, public health education, and healthcare system improvements. Special attention should be given to low-SDI countries and vulnerable populations to prevent structural inequalities from stalling or reversing progress in global CVD prevention.

## Limitations

This study has several limitations that should be considered. First, the analysis is based on GBD 2021 data, which, despite standardization and corrections, may still be affected by incomplete reporting and misclassification, especially in low- and middle-income countries. Secondly, although we focused on CVD burden attributable to high trans-fat intake, diet is influenced by multiple interacting factors, and it remains difficult to fully separate the independent effect of TFA consumption. Thirdly, our study targeted adults aged 55 and older, a group with high CVD risk. While this focus is relevant for public health, the findings may not be directly applicable to younger populations. Fourth, future projections rely on historical trends using the Bayesian Age-Period-Cohort model, which cannot account for unexpected changes such as new policies, technological shifts, or health crises, leading to uncertainties. Fourth, this study relies on the BAPC model to project future burden based on historical trends; however, this model cannot account for unexpected changes such as the introduction of new policies, technological shifts, or health crises, leading to uncertainties in the projection results. In terms of the model application itself, its limitations are mainly reflected in two aspects: first, bias from the trend continuity assumption—the BAPC model’s projections fundamentally depend on the premise that “historical age-period-cohort effect trends will continue”. The emergence of unexpected factors (e.g., sudden tightening of TFA bans in SDI regions) may cause discrepancies between the projected results and the actual burden. Although this study tested some scenarios through sensitivity analyses, it still cannot cover the impacts of all unknown variables. Subjectivity in prior selection—prior specifications (e.g., random walk order) under the Bayesian framework involve a certain degree of subjectivity. While this study determined priors by referencing previous relevant research and verified the robustness of results via sensitivity analyses, for regions with extremely sparse data, the higher weight of prior information may mask the unique burden change trends of these regions.

Finally, health inequality was assessed using SDI, a national-level indicator that may overlook regional or within-country disparities in TFA exposure and health outcomes.

## Conclusion

In summary, the global burden of CVD attributable to high TFA intake has declined significantly over the past three decades, largely due to effective policies and public health interventions in high-SDI countries. However, the burden remains high in low- and middle-SDI regions, where regulatory gaps and health inequalities persist. Future projections indicate continued progress, but growing uncertainties related to demographic changes, metabolic risks, and uneven policy enforcement highlight the need for global action.

## Data Availability

Publicly available datasets were analyzed in this study. The data comes from a public database, can through this link: https://vizhub.healthdata.org/gbd-results/ for the relevant data.

## Footnotes

1
https://vizhub.healthdata.org/gbd
