# Correction: Impact of diets high in trans-fatty acids on cardiovascular diseases in adults aged 55 and older: insights from the Global Burden of Disease 2021 data

**DOI:** 10.3389/fnut.2026.1824926

**Published:** 2026-04-08

**Authors:** Jishi Ye, Yu Liu, Juan Ren, Ruolan Wu, Jingli Chen, Rong Xiang, Yifan Jia

**Affiliations:** 1 Department of Pain, Renmin Hospital of Wuhan University, Wuhan, Hubei, China; 2 Department of Otolaryngology-Head and Neck Surgery, Renmin Hospital of Wuhan University, Wuhan, Hubei, China; 3 Department of Intensive Care Unit, The Central Hospital of Wuhan, Tongji Medical College, Huazhong University of Science and Technology, Wuhan, Hubei, China; 4 Department of Respiratory Medicine, General Hospital of Central Theater Command, Wuhan, China; 5 Department of Anesthesiology, The Central Hospital of Wuhan, Tongji Medical College, Huazhong University of Science and Technology, Wuhan, Hubei, China

**Keywords:** cardiovascular disease, Global Burden of Disease, health inequality, high trans-fatty acid, socioeconomic disparities

In the published article, there was a mistake in the author-affiliation linking. The author, Yu Liu, was incorrectly assigned affiliation 1, “Department of Pain, Renmin Hospital of Wuhan University, Wuhan, Hubei, China”. Their correctly assigned affiliation should be affiliation 3 “Department of Intensive Care Unit, The Central Hospital of Wuhan, Tongji Medical College, Huazhong University of Science and Technology, Wuhan, Hubei, China.”

The original version of this article has been updated.

