# Supplementary material for: Impact of diets high in trans-fatty acids on cardiovascular diseases in adults aged 55 and older: insights from the Global Burden of Disease 2021 data
Source: Front Nutr. 2026 Mar 6;13:1681274. doi: 10.3389/fnut.2026.1681274 (PMC13002384; doi:10.3389/fnut.2026.1681274)
Supplement: Supplementary file 1 [file Table_1.docx]

+

| **Table S1 Cardiovascular Disease Mortality Burden and Age-Standardized Mortality Rate Trends Attributable to High TFA Intake Among Adults Aged 55 and Older between 1990 and 2021 at the national level** | | | | | | |
| --- | --- | --- | --- | --- | --- | --- |
| **Measure** | **Location** | **Number 1990-95%UI** | **ASR 1990** | **Number 2021-95%UI** | **ASR 2021** | **EAPC (95% CI)** |
| Deaths | China | 2717.8 (274.3, 5779.4) | 1.9 (0.2, 4) | 4790 (492.1, 11006) | 1.3 (0.1, 2.9) | -2.0 (-2.5,-1.5) |
| Deaths | Cambodia | 1.4 (0.1, 3.7) | 0.2 (0, 0.5) | 1.3 (0.1, 3.6) | 0.1 (0, 0.2) | -5.3 (-5.9,-4.6) |
| Deaths | Taiwan (Province of China) | 5.4 (0.6, 13.1) | 0.2 (0, 0.5) | 3.7 (0.4, 9.8) | 0 (0, 0.1) | -5.5 (-6.0,-5.0) |
| Deaths | Lao People's Democratic Republic | 1.3 (0.1, 3.6) | 0.4 (0, 1.1) | 0.7 (0, 2.1) | 0.1 (0, 0.3) | -6.0 (-6.7,-5.4) |
| Deaths | Democratic People's Republic of Korea | 7.7 (0.7, 20) | 0.3 (0, 0.8) | 8.6 (0.7, 23.9) | 0.2 (0, 0.4) | -2.9 (-3.4,-2.4) |
| Deaths | Indonesia | 36.9 (2.9, 90.2) | 0.2 (0, 0.6) | 45.5 (3.5, 123.5) | 0.1 (0, 0.3) | -3.6 (-4.1,-3.0) |
| Deaths | Philippines | 4.4 (0.3, 10.4) | 0.1 (0, 0.2) | 6.1 (0.3, 16.7) | 0 (0, 0.1) | -3.8 (-4.4,-3.1) |
| Deaths | Maldives | 0 (0, 0.1) | 0.2 (0, 0.7) | 0 (0, 0.1) | 0 (0, 0.1) | -6.7 (-7.2,-6.1) |
| Deaths | Malaysia | 55.9 (5.4, 117) | 3.8 (0.4, 7.9) | 66.1 (6.9, 156.7) | 1.3 (0.1, 3.2) | -4.6 (-5.2,-4.0) |
| Deaths | Thailand | 1.5 (0, 4.2) | 0 (0, 0.1) | 0 (0, 0) | 0 (0, 0) | NA (NA,NA) |
| Deaths | Viet Nam | 8.4 (0.7, 21.4) | 0.1 (0, 0.3) | 7.7 (0.5, 21.6) | 0 (0, 0.1) | -4.1 (-4.6,-3.5) |
| Deaths | Myanmar | 12.1 (1.1, 31.7) | 0.3 (0, 0.8) | 6.6 (0.4, 20.3) | 0.1 (0, 0.2) | -5.9 (-6.5,-5.2) |
| Deaths | Sri Lanka | 4.1 (0.4, 10.2) | 0.2 (0, 0.6) | 2.8 (0.2, 8.5) | 0.1 (0, 0.2) | -5.5 (-6.2,-4.9) |
| Deaths | Timor-Leste | 0.1 (0, 0.2) | 0.2 (0, 0.5) | 0.1 (0, 0.4) | 0.1 (0, 0.2) | -3.2 (-3.7,-2.7) |
| Deaths | Fiji | 0.4 (0, 1.2) | 0.8 (0.1, 2.1) | 0.4 (0, 1) | 0.3 (0, 0.7) | -4.7 (-5.3,-4.2) |
| Deaths | Marshall Islands | 0 (0, 0.1) | 0.7 (0.1, 2) | 0 (0, 0) | 0.2 (0, 0.7) | -4.7 (-5.3,-4.2) |
| Deaths | Papua New Guinea | 1 (0.1, 2.9) | 0.3 (0, 1) | 1.2 (0.1, 3.5) | 0.1 (0, 0.4) | -4.0 (-4.7,-3.4) |
| Deaths | Solomon Islands | 0.2 (0, 0.5) | 0.8 (0.1, 2) | 0.2 (0, 0.4) | 0.3 (0, 0.8) | -4.1 (-4.6,-3.7) |
| Deaths | Micronesia (Federated States of) | 0.1 (0, 0.2) | 0.7 (0.1, 1.9) | 0 (0, 0.1) | 0.3 (0, 0.7) | -4.8 (-5.3,-4.2) |
| Deaths | Kiribati | 0 (0, 0.1) | 0.5 (0, 1.3) | 0 (0, 0.1) | 0.2 (0, 0.6) | -4.3 (-4.9,-3.8) |
| Deaths | Samoa | 0.1 (0, 0.2) | 0.6 (0.1, 1.4) | 0.1 (0, 0.2) | 0.3 (0, 0.7) | -3.7 (-4.2,-3.1) |
| Deaths | Vanuatu | 0.1 (0, 0.2) | 0.8 (0.1, 2.2) | 0.1 (0, 0.2) | 0.3 (0, 0.8) | -4.3 (-4.9,-3.8) |
| Deaths | Azerbaijan | 892.6 (81, 1690.8) | 102.3 (9.3, 193.9) | 982.4 (94.9, 1946.4) | 51.3 (5, 101.6) | -2.6 (-3.3,-1.8) |
| Deaths | Tonga | 0 (0, 0.1) | 0.4 (0, 1) | 0 (0, 0.1) | 0.2 (0, 0.5) | -3.5 (-4.1,-2.9) |
| Deaths | Kazakhstan | 15.8 (1.7, 38.2) | 0.8 (0.1, 1.8) | 1 (0, 3.2) | 0 (0, 0.1) | -14.4 (-16.4,-12.5) |
| Deaths | Armenia | 20 (1.7, 44.1) | 4.1 (0.4, 9.1) | 12.5 (1.1, 29.6) | 1.6 (0.1, 3.8) | -4.3 (-5.1,-3.4) |
| Deaths | Georgia | 279.9 (28.4, 583.9) | 25.2 (2.6, 52.6) | 18.7 (1.6, 42.8) | 1.8 (0.2, 4.1) | -11.0 (-12.3,-9.6) |
| Deaths | Kyrgyzstan | 19 (2.1, 41.8) | 3.7 (0.4, 8.1) | 13.1 (1.4, 30.8) | 1.6 (0.2, 3.6) | -3.2 (-4.4,-2.0) |
| Deaths | Mongolia | 7.6 (0.7, 16.9) | 4.4 (0.4, 9.7) | 4.4 (0.5, 11.1) | 1.1 (0.1, 2.8) | -5.8 (-6.7,-4.8) |
| Deaths | Turkmenistan | 17.4 (1.8, 36.5) | 5.4 (0.6, 11.2) | 14.7 (1.4, 35.4) | 2.1 (0.2, 5) | -4.8 (-5.9,-3.6) |
| Deaths | Tajikistan | 20.8 (2, 46) | 4.4 (0.4, 9.8) | 12.9 (1.3, 30.1) | 1.3 (0.1, 2.9) | -4.9 (-6.2,-3.7) |
| Deaths | Bosnia and Herzegovina | 2.3 (0.2, 5.8) | 0.3 (0, 0.8) | 0.3 (0, 1) | 0 (0, 0.1) | -11.0 (-12.6,-9.3) |
| Deaths | Uzbekistan | 7.1 (0.5, 19) | 0.4 (0, 1) | 2.8 (0.1, 8.8) | 0.1 (0, 0.2) | -7.8 (-8.9,-6.8) |
| Deaths | Croatia | 73.8 (7.9, 156.2) | 6.6 (0.7, 14) | 11.3 (1.1, 29.3) | 0.8 (0.1, 2) | -9.1 (-10.2,-8.0) |
| Deaths | Albania | 14.2 (1.5, 31.4) | 4.1 (0.4, 9) | 10.2 (0.9, 23) | 1.3 (0.1, 2.9) | -4.6 (-5.3,-3.9) |
| Deaths | Hungary | 467.4 (47, 961.3) | 18.1 (1.8, 37.3) | 0 (0, 0) | 0 (0, 0) | NA (NA,NA) |
| Deaths | Bulgaria | 117.6 (10.7, 270.5) | 5.2 (0.5, 12) | 20 (1.9, 46) | 0.8 (0.1, 1.9) | -8.6 (-9.9,-7.4) |
| Deaths | Montenegro | 4.6 (0.4, 10.2) | 4.2 (0.4, 9.4) | 2.8 (0.3, 6.6) | 1.6 (0.2, 3.8) | -4.2 (-4.9,-3.5) |
| Deaths | Czechia | 580.1 (59.3, 1178.3) | 24.5 (2.5, 49.8) | 96.3 (9.8, 213.1) | 2.8 (0.3, 6.1) | -8.8 (-9.6,-7.9) |
| Deaths | North Macedonia | 6.2 (0.6, 14.5) | 1.9 (0.2, 4.4) | 3.7 (0.3, 9.4) | 0.6 (0.1, 1.6) | -5.0 (-5.7,-4.4) |
| Deaths | Romania | 301.6 (33.3, 678.4) | 6 (0.7, 13.6) | 97.7 (8.7, 242.1) | 1.6 (0.1, 4) | -6.1 (-6.9,-5.3) |
| Deaths | Poland | 553.8 (51.4, 1248.4) | 7.2 (0.7, 16.2) | 86.3 (9.3, 196) | 0.7 (0.1, 1.6) | -9.8 (-10.9,-8.7) |
| Deaths | Slovakia | 234.6 (24.1, 510.2) | 22.6 (2.3, 49.2) | 71.9 (6.6, 157.3) | 4.4 (0.4, 9.6) | -6.8 (-7.9,-5.7) |
| Deaths | Serbia | 94.3 (8.8, 208.7) | 4.5 (0.4, 10) | 26.7 (2.6, 64.1) | 1 (0.1, 2.3) | -7.1 (-8.1,-6.1) |
| Deaths | Estonia | 163.2 (13.9, 326.2) | 45.5 (3.9, 91) | 36.9 (3.5, 81.8) | 8.4 (0.8, 18.7) | -6.3 (-7.2,-5.5) |
| Deaths | Slovenia | 91.7 (7.6, 184.2) | 21.3 (1.8, 42.8) | 0 (0, 0) | 0 (0, 0) | NA (NA,NA) |
| Deaths | Lithuania | 238.2 (22.9, 507.1) | 30.1 (2.9, 64) | 0 (0, 0) | 0 (0, 0) | NA (NA,NA) |
| Deaths | Belarus | 17.8 (1.5, 44.6) | 0.8 (0.1, 1.9) | 0.9 (0, 3.2) | 0 (0, 0.1) | -14.5 (-16.6,-12.4) |
| Deaths | Russian Federation | 1661 (154.2, 3665) | 5.3 (0.5, 11.6) | 384.6 (37.7, 943.1) | 0.9 (0.1, 2.2) | -8.0 (-9.2,-6.8) |
| Deaths | Latvia | 690 (57.8, 1300.1) | 110.4 (9.2, 207.9) | 0 (0, 0) | 0 (0, 0) | NA (NA,NA) |
| Deaths | Republic of Moldova | 34.1 (3.7, 78.2) | 4.4 (0.5, 10.1) | 32.5 (2.9, 72.4) | 3.1 (0.3, 6.9) | -1.8 (-2.9,-0.7) |
| Deaths | Brunei Darussalam | 0.4 (0, 0.9) | 2.4 (0.2, 5.6) | 0.6 (0.1, 1.4) | 1 (0.1, 2.4) | -2.7 (-3.0,-2.3) |
| Deaths | Ukraine | 39.7 (2.8, 110.2) | 0.3 (0, 0.9) | 6.2 (0.1, 22.7) | 0 (0, 0.2) | -9.5 (-11.1,-7.9) |
| Deaths | Republic of Korea | 378.1 (30.4, 741.3) | 7.6 (0.6, 14.9) | 726.2 (56.2, 1489.9) | 4.3 (0.3, 8.9) | -2.6 (-3.0,-2.2) |
| Deaths | Japan | 111.9 (13.7, 260.4) | 0.4 (0, 0.9) | 72.9 (8.6, 175.7) | 0.1 (0, 0.3) | -3.9 (-4.3,-3.5) |
| Deaths | New Zealand | 212 (21.1, 400.7) | 32.2 (3.2, 60.9) | 79 (7.4, 163.1) | 5.5 (0.5, 11.4) | -6.9 (-7.4,-6.3) |
| Deaths | Singapore | 10.3 (1.1, 23.3) | 2.8 (0.3, 6.4) | 4.2 (0.4, 9.5) | 0.3 (0, 0.6) | -9.4 (-10.4,-8.4) |
| Deaths | Andorra | 0.3 (0, 0.6) | 2.8 (0.3, 6) | 0.2 (0, 0.4) | 0.7 (0.1, 1.6) | -5.2 (-5.7,-4.6) |
| Deaths | Australia | 1374.3 (112.6, 2667.9) | 41.9 (3.4, 81.3) | 553.3 (53.9, 1116.1) | 7.5 (0.7, 15.1) | -6.5 (-6.9,-6.1) |
| Deaths | Belgium | 492 (39.4, 969.3) | 18.8 (1.5, 37) | 115.6 (13.8, 244) | 3.1 (0.4, 6.5) | -6.7 (-7.3,-6.1) |
| Deaths | Austria | 33.4 (3.2, 77.2) | 1.7 (0.2, 4) | 0 (0, 0) | 0 (0, 0) | NA (NA,NA) |
| Deaths | Denmark | 293.8 (29.3, 608.6) | 22.5 (2.2, 46.7) | 0 (0, 0) | 0 (0, 0) | NA (NA,NA) |
| Deaths | France | 758.7 (78.9, 1585.8) | 5.5 (0.6, 11.4) | 297.3 (28.7, 663.8) | 1.3 (0.1, 3) | -5.6 (-6.1,-5.0) |
| Deaths | Greece | 214.9 (16.9, 446.6) | 8 (0.6, 16.7) | 48.7 (4.8, 108.8) | 1.3 (0.1, 3) | -7.9 (-8.8,-7.0) |
| Deaths | Cyprus | 9.5 (1, 20.1) | 6.8 (0.7, 14.4) | 3.8 (0.4, 8.7) | 1.1 (0.1, 2.5) | -7.4 (-8.1,-6.8) |
| Deaths | Finland | 145.9 (14.7, 303) | 12.3 (1.2, 25.5) | 27.9 (2.5, 64.6) | 1.4 (0.1, 3.2) | -8.8 (-9.7,-8.0) |
| Deaths | Germany | 6945.2 (596, 13552.1) | 33.1 (2.8, 64.6) | 1977.7 (209.9, 4088.7) | 6.3 (0.7, 13) | -6.3 (-6.8,-5.7) |
| Deaths | Iceland | 3.5 (0.3, 7.4) | 7.3 (0.6, 15.5) | 0 (0, 0) | 0 (0, 0) | NA (NA,NA) |
| Deaths | Italy | 22.3 (1.8, 56.6) | 0.1 (0, 0.4) | 12.8 (1.2, 36) | 0.1 (0, 0.2) | -3.7 (-4.1,-3.3) |
| Deaths | Ireland | 35.9 (3.7, 80.8) | 5.3 (0.5, 11.9) | 4 (0.4, 9.6) | 0.3 (0, 0.7) | -11.1 (-12.1,-10.0) |
| Deaths | Israel | 8 (0.8, 20.2) | 1 (0.1, 2.5) | 1.9 (0.1, 5.2) | 0.1 (0, 0.3) | -9.0 (-9.7,-8.4) |
| Deaths | Norway | 96.9 (10.4, 207) | 9 (1, 19.1) | 0 (0, 0) | 0 (0, 0) | NA (NA,NA) |
| Deaths | Malta | 5.6 (0.6, 12.3) | 7.7 (0.8, 17) | 2.1 (0.2, 4.6) | 1.3 (0.1, 2.9) | -7.0 (-7.6,-6.4) |
| Deaths | Portugal | 138.8 (12.7, 302.9) | 5.7 (0.5, 12.4) | 25.4 (2.7, 54.9) | 0.7 (0.1, 1.4) | -8.9 (-9.9,-8.0) |
| Deaths | Netherlands | 669.9 (64.5, 1345) | 20.2 (1.9, 40.6) | 278 (24.9, 563.7) | 4.8 (0.4, 9.6) | -5.5 (-6.0,-5.0) |
| Deaths | Spain | 12.9 (0.9, 35.7) | 0.1 (0, 0.4) | 4.1 (0.3, 11.5) | 0 (0, 0.1) | -6.6 (-7.4,-5.9) |
| Deaths | Luxembourg | 5.4 (0.6, 11.3) | 5.8 (0.6, 12.1) | 1.5 (0.2, 3.2) | 0.8 (0.1, 1.8) | -7.3 (-7.9,-6.7) |
| Deaths | Switzerland | 340.7 (35.6, 697.9) | 20.2 (2.1, 41.3) | 73.7 (7, 160.6) | 2.5 (0.2, 5.5) | -8.0 (-8.7,-7.3) |
| Deaths | Sweden | 38.7 (3.8, 94.7) | 1.6 (0.2, 4) | 5.6 (0.5, 13.9) | 0.2 (0, 0.4) | -8.7 (-9.4,-7.9) |
| Deaths | United Kingdom | 3309.7 (369.5, 6592) | 22.3 (2.5, 44.4) | 814.4 (81.3, 1641.1) | 3.9 (0.4, 7.8) | -6.4 (-6.8,-5.9) |
| Deaths | Argentina | 46.8 (4.3, 110.7) | 0.8 (0.1, 2) | 30.8 (3.1, 69.7) | 0.3 (0, 0.7) | -2.8 (-2.9,-2.6) |
| Deaths | Chile | 38 (3.9, 82.2) | 2.3 (0.2, 4.9) | 0 (0, 0) | 0 (0, 0) | NA (NA,NA) |
| Deaths | Uruguay | 3.9 (0.4, 9.4) | 0.6 (0.1, 1.4) | 0.8 (0.1, 2.3) | 0.1 (0, 0.3) | -7.3 (-8.0,-6.6) |
| Deaths | United States of America | 41074.9 (3814.3, 77983.6) | 78.3 (7.3, 148.6) | 0 (0, 0) | 0 (0, 0) | NA (NA,NA) |
| Deaths | Canada | 2907.4 (238, 5403.5) | 53.3 (4.4, 99) | 0 (0, 0) | 0 (0, 0) | NA (NA,NA) |
| Deaths | Bahamas | 0 (0, 0.1) | 0.1 (0, 0.3) | 0 (0, 0) | 0 (0, 0.1) | -7.8 (-8.5,-7.1) |
| Deaths | Belize | 0 (0, 0.1) | 0.1 (0, 0.4) | 0 (0, 0) | 0 (0, 0.1) | -8.6 (-9.7,-7.6) |
| Deaths | Antigua and Barbuda | 0 (0, 0) | 0.2 (0, 0.5) | 0 (0, 0) | 0 (0, 0.1) | -9.0 (-9.7,-8.3) |
| Deaths | Dominica | 0 (0, 0.1) | 0.2 (0, 0.5) | 0 (0, 0) | 0 (0, 0.1) | -8.0 (-8.7,-7.2) |
| Deaths | Cuba | 4.3 (0.4, 11.6) | 0.3 (0, 0.7) | 1.3 (0, 4) | 0 (0, 0.1) | -7.8 (-8.6,-7.1) |
| Deaths | Barbados | 0.1 (0, 0.2) | 0.2 (0, 0.5) | 0 (0, 0.1) | 0 (0, 0.1) | -8.7 (-9.5,-7.9) |
| Deaths | Grenada | 0 (0, 0.1) | 0.2 (0, 0.5) | 0 (0, 0) | 0 (0, 0.1) | -8.7 (-9.5,-7.9) |
| Deaths | Haiti | 1.3 (0.1, 3.5) | 0.2 (0, 0.6) | 0.6 (0, 1.8) | 0 (0, 0.2) | -6.5 (-7.2,-5.8) |
| Deaths | Dominican Republic | 0.5 (0, 1.4) | 0.1 (0, 0.2) | 0.4 (0, 1.2) | 0 (0, 0.1) | -5.6 (-6.5,-4.8) |
| Deaths | Saint Lucia | 0 (0, 0.1) | 0.2 (0, 0.4) | 0 (0, 0) | 0 (0, 0) | -9.2 (-10.0,-8.5) |
| Deaths | Guyana | 0.1 (0, 0.4) | 0.2 (0, 0.6) | 0 (0, 0.1) | 0 (0, 0.1) | -7.4 (-8.1,-6.7) |
| Deaths | Suriname | 0.1 (0, 0.2) | 0.2 (0, 0.5) | 0 (0, 0.1) | 0 (0, 0.1) | -7.6 (-8.3,-6.8) |
| Deaths | Jamaica | 0.3 (0, 0.8) | 0.1 (0, 0.3) | 0.1 (0, 0.3) | 0 (0, 0.1) | -6.3 (-7.0,-5.7) |
| Deaths | Saint Vincent and the Grenadines | 0 (0, 0.1) | 0.2 (0, 0.6) | 0 (0, 0) | 0 (0, 0.1) | -7.8 (-8.5,-7.2) |
| Deaths | Mexico | 1322.7 (133.1, 2625.3) | 19 (1.9, 37.8) | 2494.2 (248.8, 5040.5) | 11.6 (1.2, 23.4) | -2.5 (-2.9,-2.0) |
| Deaths | Ecuador | 150.1 (13.8, 280.1) | 17.5 (1.6, 32.6) | 250.5 (24.4, 515.5) | 9.1 (0.9, 18.6) | -2.8 (-3.5,-2.1) |
| Deaths | Trinidad and Tobago | 0.3 (0, 0.8) | 0.2 (0, 0.6) | 0.1 (0, 0.3) | 0 (0, 0.1) | -8.8 (-9.7,-7.8) |
| Deaths | Bolivia (Plurinational State of) | 44.8 (4.3, 91.7) | 8.5 (0.8, 17.5) | 46.9 (4.8, 103.6) | 3 (0.3, 6.7) | -3.9 (-4.2,-3.6) |
| Deaths | Peru | 179.9 (15.8, 362.5) | 9.1 (0.8, 18.4) | 192.3 (15.8, 412.5) | 3.4 (0.3, 7.4) | -4.0 (-4.6,-3.5) |
| Deaths | Costa Rica | 57.5 (5.7, 113.3) | 20.2 (2, 39.8) | 24.1 (2.3, 52.2) | 2.5 (0.2, 5.5) | -8.3 (-9.1,-7.4) |
| Deaths | Colombia | 232.1 (24, 477.4) | 8.1 (0.8, 16.6) | 127.6 (12.8, 279) | 1.3 (0.1, 2.9) | -7.7 (-8.6,-6.8) |
| Deaths | Guatemala | 6.6 (0.6, 14.8) | 1.1 (0.1, 2.6) | 8.8 (0.9, 22.1) | 0.5 (0.1, 1.2) | -3.6 (-4.3,-3.0) |
| Deaths | El Salvador | 43.5 (4.3, 88.6) | 8.9 (0.9, 18.1) | 33.3 (3.9, 70.9) | 3.3 (0.4, 6.9) | -4.3 (-4.9,-3.7) |
| Deaths | Honduras | 24.3 (2.5, 51.9) | 7.3 (0.7, 15.6) | 45.4 (3.9, 97.8) | 4.3 (0.4, 9.3) | -2.7 (-3.3,-2.0) |
| Deaths | Panama | 19.4 (1.7, 40.8) | 8 (0.7, 16.8) | 13.2 (1.1, 29.7) | 1.8 (0.2, 4) | -5.7 (-6.2,-5.2) |
| Deaths | Nicaragua | 15.2 (1.5, 31.2) | 6.1 (0.6, 12.6) | 17.8 (1.8, 39.9) | 2.2 (0.2, 4.9) | -4.2 (-4.9,-3.4) |
| Deaths | Paraguay | 28.9 (3, 58.6) | 8 (0.8, 16.2) | 30.2 (3, 66) | 3 (0.3, 6.7) | -3.6 (-4.0,-3.3) |
| Deaths | Venezuela (Bolivarian Republic of) | 285.6 (29.6, 573.1) | 18.2 (1.9, 36.6) | 226.3 (21.3, 501) | 4.3 (0.4, 9.6) | -6.8 (-7.8,-5.8) |
| Deaths | Brazil | 1395 (158.9, 2852) | 9.4 (1.1, 19.3) | 1114.9 (106.3, 2250.9) | 2.6 (0.2, 5.2) | -4.9 (-5.4,-4.5) |
| Deaths | Algeria | 102.2 (8.8, 232.2) | 4.9 (0.4, 11.1) | 51.4 (4.9, 129.8) | 0.8 (0.1, 2.1) | -8.0 (-9.1,-6.8) |
| Deaths | Bahrain | 1.3 (0.1, 3.1) | 4.8 (0.5, 10.9) | 3.3 (0.3, 7.2) | 2 (0.2, 4.4) | -3.7 (-4.7,-2.6) |
| Deaths | Lebanon | 13.6 (1.3, 30.1) | 3.6 (0.3, 7.9) | 26.5 (2.6, 57.6) | 2.7 (0.3, 5.9) | -0.3 (-0.7,0.2) |
| Deaths | Jordan | 6.2 (0.6, 13.8) | 2.9 (0.3, 6.4) | 22.8 (2.3, 52.9) | 1.8 (0.2, 4.2) | -1.5 (-2.4,-0.6) |
| Deaths | Iran (Islamic Republic of) | 2851.2 (278.8, 5450.4) | 61.8 (6, 118.2) | 4686 (439.2, 8971.4) | 36.1 (3.4, 69.1) | -2.0 (-2.4,-1.6) |
| Deaths | Libya | 8.2 (0.8, 18.3) | 2.7 (0.3, 5.9) | 31.4 (3.5, 71) | 3.8 (0.4, 8.5) | 2.1 (1.2,3.1) |
| Deaths | Iraq | 56.1 (6, 123.5) | 4.4 (0.5, 9.8) | 199 (18.4, 446.3) | 5.1 (0.5, 11.5) | 0.4 (-0.2,1.1) |
| Deaths | Egypt | 4951.6 (448, 9188.6) | 110.7 (10, 205.5) | 10612.9 (818.6, 20525.6) | 95.9 (7.4, 185.5) | -0.2 (-0.5,0.1) |
| Deaths | Kuwait | 2.7 (0.3, 6.3) | 2.9 (0.3, 6.9) | 10.3 (0.9, 23.9) | 2.2 (0.2, 5.1) | -0.3 (-1.4,0.7) |
| Deaths | Morocco | 114.7 (11.2, 248.1) | 4.9 (0.5, 10.6) | 342.6 (31.9, 745.7) | 5.7 (0.5, 12.4) | 0.9 (0.2,1.6) |
| Deaths | Oman | 5.2 (0.5, 11.7) | 5.2 (0.4, 11.7) | 10.1 (0.9, 21) | 3.2 (0.3, 6.7) | -0.7 (-1.5,0.1) |
| Deaths | Saudi Arabia | 16.5 (1.3, 37.6) | 1.9 (0.2, 4.2) | 0 (0, 0) | 0 (0, 0) | NA (NA,NA) |
| Deaths | Syrian Arab Republic | 48.8 (4.8, 106.7) | 5.5 (0.5, 12.1) | 151 (16, 343.6) | 6.4 (0.7, 14.5) | 0.6 (0.1,1.2) |
| Deaths | Qatar | 0.7 (0.1, 1.5) | 4.2 (0.4, 9.2) | 2.1 (0.2, 4.8) | 1.4 (0.1, 3.1) | -3.9 (-5.0,-2.7) |
| Deaths | Palestine | 6.7 (0.6, 16.3) | 4.6 (0.4, 11.2) | 15.3 (1.5, 33) | 3.6 (0.4, 7.7) | -0.4 (-1.3,0.4) |
| Deaths | Tunisia | 4.3 (0.4, 10.7) | 0.5 (0, 1.2) | 2.6 (0.1, 7.6) | 0.1 (0, 0.3) | -7.0 (-8.0,-6.0) |
| Deaths | United Arab Emirates | 3.7 (0.4, 8.2) | 6.6 (0.7, 14.7) | 8.6 (0.8, 19.3) | 1.2 (0.1, 2.7) | -5.2 (-5.7,-4.7) |
| Deaths | Afghanistan | 69 (7.1, 170.9) | 5.7 (0.6, 14.1) | 75.8 (7.5, 169.7) | 6.2 (0.6, 13.8) | 0.8 (0.1,1.4) |
| Deaths | Bhutan | 6.8 (0.6, 13.8) | 17.1 (1.4, 34.7) | 12.3 (1, 25.6) | 12.3 (1, 25.7) | -1.5 (-1.7,-1.3) |
| Deaths | Yemen | 38.8 (3, 94.8) | 4.8 (0.4, 11.8) | 120.7 (11.8, 276.9) | 5.4 (0.5, 12.3) | 0.6 (-0.1,1.3) |
| Deaths | Turkey | 8.7 (0.6, 24.6) | 0.1 (0, 0.4) | 6.4 (0.2, 19.5) | 0 (0, 0.1) | -5.5 (-6.2,-4.7) |
| Deaths | Bangladesh | 1590.1 (141.7, 3194.1) | 21 (1.9, 42.2) | 2631.7 (244.4, 5415.1) | 11.3 (1, 23.2) | -2.5 (-2.8,-2.2) |
| Deaths | Nepal | 326.2 (29.3, 644.5) | 21.2 (1.9, 41.9) | 542.8 (48.9, 1111.6) | 13.7 (1.2, 28.1) | -1.7 (-1.8,-1.5) |
| Deaths | India | 18961.1 (2057.2, 37334) | 24.7 (2.7, 48.7) | 31146.3 (2971.3, 61199.6) | 15.5 (1.5, 30.4) | -2.2 (-2.6,-1.9) |
| Deaths | Angola | 1.3 (0.1, 3.6) | 0.2 (0, 0.6) | 1.7 (0.1, 4.5) | 0.1 (0, 0.2) | -3.9 (-4.4,-3.3) |
| Deaths | Pakistan | 1922.4 (218.3, 3897.5) | 21.1 (2.4, 42.7) | 3673.7 (347.5, 7743.9) | 18.5 (1.8, 39.1) | -1.0 (-1.3,-0.7) |
| Deaths | Congo | 0.5 (0, 1.4) | 0.3 (0, 0.8) | 0.5 (0, 1.5) | 0.1 (0, 0.3) | -4.3 (-4.9,-3.7) |
| Deaths | Central African Republic | 0.5 (0, 1.4) | 0.3 (0, 0.7) | 0.3 (0, 1) | 0.1 (0, 0.3) | -4.2 (-4.8,-3.6) |
| Deaths | Equatorial Guinea | 0.1 (0, 0.2) | 0.3 (0, 0.7) | 0.1 (0, 0.3) | 0.1 (0, 0.3) | -3.9 (-4.5,-3.4) |
| Deaths | Democratic Republic of the Congo | 5.5 (0.5, 15.4) | 0.2 (0, 0.6) | 5 (0.3, 13.3) | 0.1 (0, 0.2) | -4.1 (-4.7,-3.5) |
| Deaths | Comoros | 0 (0, 0.1) | 0.1 (0, 0.3) | 0 (0, 0.1) | 0.1 (0, 0.2) | -3.5 (-4.0,-3.0) |
| Deaths | Gabon | 0.3 (0, 0.7) | 0.3 (0, 0.7) | 0.2 (0, 0.5) | 0.1 (0, 0.3) | -4.1 (-4.8,-3.5) |
| Deaths | Burundi | 0.7 (0.1, 2) | 0.2 (0, 0.5) | 0.5 (0, 1.5) | 0.1 (0, 0.2) | -5.1 (-5.8,-4.4) |
| Deaths | Eritrea | 0.2 (0, 0.6) | 0.1 (0, 0.3) | 0.3 (0, 0.8) | 0.1 (0, 0.2) | -3.1 (-3.7,-2.6) |
| Deaths | Kenya | 1.7 (0.2, 4) | 0.1 (0, 0.3) | 3.3 (0.3, 8.2) | 0.1 (0, 0.2) | -1.7 (-2.3,-1.0) |
| Deaths | Ethiopia | 6 (0.6, 15.5) | 0.2 (0, 0.5) | 4.7 (0.4, 12.3) | 0.1 (0, 0.2) | -4.3 (-4.7,-3.8) |
| Deaths | Djibouti | 0 (0, 0.1) | 0.1 (0, 0.3) | 0.1 (0, 0.2) | 0.1 (0, 0.2) | -2.8 (-3.4,-2.2) |
| Deaths | Madagascar | 1.2 (0.1, 3.3) | 0.1 (0, 0.4) | 1.3 (0.1, 4) | 0.1 (0, 0.2) | -3.8 (-4.4,-3.1) |
| Deaths | Malawi | 0.8 (0.1, 2) | 0.1 (0, 0.3) | 0.8 (0.1, 2.2) | 0.1 (0, 0.2) | -3.2 (-4.0,-2.4) |
| Deaths | Mozambique | 0.6 (0, 1.5) | 0.1 (0, 0.2) | 0.7 (0, 1.9) | 0 (0, 0.1) | -2.2 (-2.7,-1.6) |
| Deaths | Seychelles | 0 (0, 0.1) | 0.3 (0, 0.7) | 0 (0, 0) | 0 (0, 0.1) | -6.6 (-7.2,-6.0) |
| Deaths | United Republic of Tanzania | 2.2 (0.2, 5.7) | 0.1 (0, 0.3) | 3.3 (0.2, 9.6) | 0.1 (0, 0.2) | -2.4 (-3.0,-1.8) |
| Deaths | Mauritius | 0.5 (0, 1.2) | 0.4 (0, 1) | 0.2 (0, 0.6) | 0.1 (0, 0.2) | -8.1 (-9.0,-7.2) |
| Deaths | Rwanda | 0.8 (0.1, 2) | 0.2 (0, 0.4) | 0.5 (0, 1.4) | 0 (0, 0.1) | -5.9 (-6.7,-5.1) |
| Deaths | Somalia | 0.4 (0, 1.1) | 0.1 (0, 0.3) | 0.5 (0, 1.4) | 0 (0, 0.2) | -3.4 (-3.9,-2.8) |
| Deaths | Zambia | 0.5 (0, 1.3) | 0.1 (0, 0.3) | 0.8 (0, 2.3) | 0.1 (0, 0.2) | -2.3 (-2.8,-1.7) |
| Deaths | Botswana | 0 (0, 0.1) | 0 (0, 0.1) | 0 (0, 0) | 0 (0, 0) | -6.2 (-6.9,-5.4) |
| Deaths | Eswatini | 0 (0, 0) | 0 (0, 0.1) | 0 (0, 0) | 0 (0, 0) | -4.8 (-5.6,-4.0) |
| Deaths | Namibia | 0 (0, 0.1) | 0 (0, 0.1) | 0 (0, 0) | 0 (0, 0) | -5.6 (-6.5,-4.6) |
| Deaths | Uganda | 1.4 (0.1, 3.5) | 0.1 (0, 0.3) | 1.4 (0.1, 4) | 0.1 (0, 0.2) | -4.1 (-4.9,-3.3) |
| Deaths | South Africa | 0.8 (0.1, 2.5) | 0 (0, 0.1) | 0 (0, 0) | 0 (0, 0) | NA (NA,NA) |
| Deaths | Lesotho | 0 (0, 0.1) | 0 (0, 0) | 0 (0, 0) | 0 (0, 0) | -3.7 (-4.5,-2.9) |
| Deaths | Zimbabwe | 0.1 (0, 0.4) | 0 (0, 0.1) | 0.1 (0, 0.3) | 0 (0, 0) | -4.3 (-5.4,-3.3) |
| Deaths | Burkina Faso | 1.1 (0.1, 3) | 0.2 (0, 0.4) | 1.3 (0.1, 3.5) | 0.1 (0, 0.2) | -2.7 (-3.3,-2.1) |
| Deaths | Benin | 0.5 (0, 1.2) | 0.2 (0, 0.4) | 0.5 (0, 1.4) | 0.1 (0, 0.2) | -3.6 (-4.1,-3.1) |
| Deaths | Cabo Verde | 0.1 (0, 0.2) | 0.2 (0, 0.4) | 0.1 (0, 0.2) | 0.1 (0, 0.3) | -2.5 (-3.3,-1.6) |
| Deaths | Cameroon | 1 (0.1, 2.9) | 0.1 (0, 0.4) | 1.6 (0.1, 4.7) | 0.1 (0, 0.2) | -2.6 (-3.6,-1.7) |
| Deaths | Chad | 0.8 (0.1, 2.1) | 0.2 (0, 0.4) | 0.8 (0, 2.3) | 0.1 (0, 0.2) | -3.4 (-4.1,-2.7) |
| Deaths | Côte d'Ivoire | 1.2 (0.1, 3.2) | 0.2 (0, 0.5) | 1.6 (0.1, 4.4) | 0.1 (0, 0.2) | -3.5 (-4.2,-2.7) |
| Deaths | Gambia | 0.1 (0, 0.3) | 0.2 (0, 0.6) | 0.2 (0, 0.6) | 0.1 (0, 0.4) | -2.6 (-3.2,-2.0) |
| Deaths | Ghana | 2.4 (0.2, 5.8) | 0.2 (0, 0.6) | 2.1 (0.1, 5.9) | 0.1 (0, 0.2) | -4.9 (-5.6,-4.2) |
| Deaths | Guinea | 1 (0.1, 2.6) | 0.2 (0, 0.5) | 0.9 (0.1, 2.6) | 0.1 (0, 0.3) | -2.8 (-3.4,-2.1) |
| Deaths | Guinea-Bissau | 0.2 (0, 0.4) | 0.3 (0, 0.7) | 0.1 (0, 0.4) | 0.1 (0, 0.4) | -3.6 (-4.2,-3.1) |
| Deaths | Mali | 0.8 (0.1, 2.3) | 0.1 (0, 0.3) | 0.8 (0.1, 2.3) | 0.1 (0, 0.2) | -3.3 (-3.7,-2.9) |
| Deaths | Liberia | 0.4 (0, 1.1) | 0.2 (0, 0.6) | 0.3 (0, 0.8) | 0.1 (0, 0.3) | -3.8 (-4.5,-3.1) |
| Deaths | Sao Tome and Principe | 0 (0, 0) | 0.2 (0, 0.4) | 0 (0, 0) | 0.1 (0, 0.2) | -2.7 (-3.4,-2.1) |
| Deaths | Mauritania | 0.4 (0, 1.2) | 0.3 (0, 0.7) | 0.4 (0, 1) | 0.1 (0, 0.3) | -4.5 (-5.1,-3.9) |
| Deaths | Sierra Leone | 0.9 (0.1, 2.2) | 0.3 (0, 0.7) | 0.7 (0, 2) | 0.1 (0, 0.3) | -3.6 (-4.3,-2.9) |
| Deaths | Senegal | 1.3 (0.1, 3.3) | 0.2 (0, 0.6) | 1.3 (0.1, 3.6) | 0.1 (0, 0.3) | -3.8 (-4.4,-3.2) |
| Deaths | Nigeria | 20.3 (1.4, 48.8) | 0.3 (0, 0.7) | 19.5 (2.1, 48.5) | 0.1 (0, 0.3) | -3.1 (-3.6,-2.6) |
| Deaths | Niger | 0.5 (0.1, 1.3) | 0.1 (0, 0.3) | 0.8 (0.1, 2) | 0.1 (0, 0.1) | -3.2 (-3.8,-2.7) |
| Deaths | Bermuda | 0 (0, 0.1) | 0.3 (0, 0.7) | 0 (0, 0) | 0 (0, 0.1) | -8.9 (-9.6,-8.2) |
| Deaths | Togo | 0.4 (0, 1.1) | 0.2 (0, 0.5) | 0.5 (0, 1.6) | 0.1 (0, 0.3) | -4.0 (-4.7,-3.4) |
| Deaths | Greenland | 2.4 (0.2, 4.7) | 43.3 (3.6, 85.3) | 2.3 (0.2, 4.3) | 16.6 (1.4, 32) | -3.1 (-3.2,-2.9) |
| Deaths | American Samoa | 0 (0, 0) | 0.4 (0, 1.1) | 0 (0, 0) | 0.2 (0, 0.5) | -3.6 (-4.2,-3.0) |
| Deaths | Cook Islands | 0 (0, 0) | 0.5 (0, 1.1) | 0 (0, 0) | 0.1 (0, 0.4) | -4.9 (-5.4,-4.4) |
| Deaths | Monaco | 0.5 (0, 1.2) | 4.9 (0.4, 10.5) | 0.2 (0, 0.4) | 1 (0.1, 2.3) | -6.3 (-6.8,-5.8) |
| Deaths | Guam | 0.1 (0, 0.2) | 0.5 (0, 1.3) | 0.1 (0, 0.2) | 0.2 (0, 0.4) | -4.2 (-4.7,-3.7) |
| Deaths | Niue | 0 (0, 0) | 0.9 (0.1, 2.2) | 0 (0, 0) | 0.3 (0, 0.8) | -4.5 (-5.0,-4.0) |
| Deaths | Palau | 0 (0, 0) | 0.7 (0.1, 1.7) | 0 (0, 0) | 0.2 (0, 0.5) | -5.0 (-5.6,-4.5) |
| Deaths | Nauru | 0 (0, 0) | 1 (0.1, 2.8) | 0 (0, 0) | 0.4 (0, 1.2) | -4.2 (-4.9,-3.4) |
| Deaths | Northern Mariana Islands | 0 (0, 0) | 0.3 (0, 0.8) | 0 (0, 0) | 0.1 (0, 0.4) | -3.8 (-4.3,-3.2) |
| Deaths | Puerto Rico | 1.1 (0.1, 3) | 0.2 (0, 0.5) | 0.3 (0, 0.8) | 0 (0, 0.1) | -8.6 (-9.5,-7.8) |
| Deaths | Saint Kitts and Nevis | 0 (0, 0) | 0.3 (0, 0.7) | 0 (0, 0) | 0 (0, 0.1) | -9.6 (-10.5,-8.6) |
| Deaths | Tokelau | 0 (0, 0) | 0.7 (0.1, 1.7) | 0 (0, 0) | 0.3 (0, 0.7) | -4.0 (-4.6,-3.4) |
| Deaths | San Marino | 0.2 (0, 0.4) | 3.3 (0.3, 7.1) | 0.1 (0, 0.1) | 0.5 (0.1, 1.2) | -6.3 (-7.0,-5.7) |
| Deaths | United States Virgin Islands | 0 (0, 0.1) | 0.2 (0, 0.5) | 0 (0, 0) | 0 (0, 0.1) | -7.4 (-8.2,-6.6) |
| Deaths | Tuvalu | 0 (0, 0) | 0.7 (0.1, 1.9) | 0 (0, 0) | 0.3 (0, 0.8) | -4.2 (-4.8,-3.6) |
| Deaths | Sudan | 80.6 (9.3, 183) | 5.4 (0.6, 12.3) | 168.8 (17, 373.8) | 5.4 (0.5, 12) | 0.3 (-0.4,1.0) |
| Deaths | South Sudan | 0.6 (0.1, 1.6) | 0.1 (0, 0.4) | 0.4 (0, 1.1) | 0.1 (0, 0.2) | -3.7 (-4.3,-3.1) |

| **Table 2 Cardiovascular Disease Mortality Burden and Age-Standardized DALYs Rate Trends Attributable to High TFA Intake Among Adults Aged 55 and Older between 1990 and 2021 at the national level** | | | | | | |
| --- | --- | --- | --- | --- | --- | --- |
| **Measure** | **Location** | **Number 1990-95%UI** | **ASR 1990** | **Number 2021-95%UI** | **ASR 2021** | **EAPC (95% CI)** |
| DALYs (Disability-Adjusted Life Years) | Taiwan (Province of China) | 107.9 (12.1, 274.9) | 3.9 (0.4, 9.9) | 62.8 (6.2, 158.8) | 0.8 (0.1, 2.1) | -6.0 (-6.5,-5.5) |
| DALYs (Disability-Adjusted Life Years) | Cambodia | 31.6 (1.9, 90.7) | 4.3 (0.3, 12.2) | 25.8 (1.5, 74.4) | 1.2 (0.1, 3.4) | -5.6 (-6.2,-4.9) |
| DALYs (Disability-Adjusted Life Years) | Malaysia | 1182.6 (115.6, 2526.6) | 80.1 (7.8, 171.2) | 1376.2 (144.9, 3417.9) | 28.1 (3, 69.7) | -4.6 (-5.2,-4.0) |
| DALYs (Disability-Adjusted Life Years) | China | 57176.1 (6001.3, 121578.1) | 39.8 (4.2, 84.7) | 79733.1 (7991.3, 181712.8) | 21 (2.1, 48) | -2.8 (-3.3,-2.3) |
| DALYs (Disability-Adjusted Life Years) | Indonesia | 830.1 (60.6, 2046.2) | 5.1 (0.4, 12.7) | 987.6 (70.6, 2829.8) | 2.4 (0.2, 6.8) | -3.7 (-4.2,-3.1) |
| DALYs (Disability-Adjusted Life Years) | Democratic People's Republic of Korea | 154.4 (14.2, 416.3) | 5.8 (0.5, 15.6) | 159.2 (13.1, 446.3) | 2.8 (0.2, 7.9) | -3.3 (-3.9,-2.8) |
| DALYs (Disability-Adjusted Life Years) | Thailand | 29.6 (0.5, 84.2) | 0.5 (0, 1.4) | 0 (0, 0) | 0 (0, 0) | NA (NA,NA) |
| DALYs (Disability-Adjusted Life Years) | Maldives | 0.9 (0.1, 2.5) | 5.8 (0.5, 16.3) | 0.4 (0, 1.2) | 0.8 (0, 2.1) | -7.9 (-8.5,-7.2) |
| DALYs (Disability-Adjusted Life Years) | Lao People's Democratic Republic | 30.8 (2.4, 90.6) | 9 (0.7, 26.4) | 14.7 (0.7, 44.3) | 1.9 (0.1, 5.7) | -6.4 (-7.1,-5.8) |
| DALYs (Disability-Adjusted Life Years) | Myanmar | 266.3 (23.6, 757.4) | 6.8 (0.6, 19.3) | 127.5 (6.1, 391.6) | 1.5 (0.1, 4.6) | -6.3 (-7.0,-5.7) |
| DALYs (Disability-Adjusted Life Years) | Philippines | 86.7 (6.7, 230.1) | 1.8 (0.1, 4.8) | 123.8 (6.1, 370) | 0.9 (0, 2.7) | -3.7 (-4.4,-3.1) |
| DALYs (Disability-Adjusted Life Years) | Timor-Leste | 1.8 (0.1, 4.9) | 4.1 (0.3, 11.3) | 2.5 (0.1, 7.1) | 1.8 (0.1, 5) | -3.7 (-4.2,-3.2) |
| DALYs (Disability-Adjusted Life Years) | Sri Lanka | 82.2 (6.7, 221.5) | 4.6 (0.4, 12.4) | 53.2 (2.3, 167.7) | 1.1 (0, 3.5) | -5.7 (-6.4,-5.1) |
| DALYs (Disability-Adjusted Life Years) | Marshall Islands | 0.4 (0, 1.2) | 16.5 (1.2, 45.6) | 0.3 (0, 1.2) | 5.9 (0.3, 19.7) | -4.3 (-4.8,-3.8) |
| DALYs (Disability-Adjusted Life Years) | Viet Nam | 159.6 (14.8, 407.2) | 2.3 (0.2, 5.8) | 141 (9.1, 434.1) | 0.8 (0.1, 2.5) | -4.2 (-4.8,-3.7) |
| DALYs (Disability-Adjusted Life Years) | Fiji | 10.4 (0.8, 30.4) | 18.4 (1.3, 54) | 8.1 (0.4, 24.3) | 5.9 (0.3, 17.8) | -4.8 (-5.3,-4.3) |
| DALYs (Disability-Adjusted Life Years) | Micronesia (Federated States of) | 1.3 (0.1, 3.4) | 16.3 (1.3, 42.3) | 0.8 (0, 2.4) | 5.9 (0.4, 18.5) | -4.4 (-5.0,-3.9) |
| DALYs (Disability-Adjusted Life Years) | Kiribati | 0.7 (0.1, 2.1) | 12.4 (1, 34.4) | 0.6 (0, 1.9) | 4.6 (0.3, 15.3) | -4.3 (-4.8,-3.7) |
| DALYs (Disability-Adjusted Life Years) | Papua New Guinea | 25.2 (1.9, 74.3) | 8.5 (0.7, 25.1) | 27.7 (1.3, 86.7) | 3.4 (0.2, 10.6) | -4.1 (-4.8,-3.5) |
| DALYs (Disability-Adjusted Life Years) | Tonga | 0.8 (0.1, 2.3) | 8.8 (0.7, 23.7) | 0.5 (0, 1.3) | 3.4 (0.3, 9.6) | -4.0 (-4.6,-3.4) |
| DALYs (Disability-Adjusted Life Years) | Vanuatu | 1.8 (0.1, 5.3) | 18.4 (1.5, 52.8) | 2 (0.1, 5.8) | 6.9 (0.5, 20.1) | -4.4 (-4.9,-3.9) |
| DALYs (Disability-Adjusted Life Years) | Samoa | 1.8 (0.1, 4.5) | 12.2 (1, 31.1) | 1.3 (0.1, 3.5) | 5.1 (0.4, 14.3) | -3.9 (-4.4,-3.3) |
| DALYs (Disability-Adjusted Life Years) | Azerbaijan | 18870.3 (1800.8, 35873.9) | 2163.5 (206.5, 4113) | 20026.7 (1967.9, 39403.5) | 1045.3 (102.7, 2056.8) | -3.0 (-3.7,-2.3) |
| DALYs (Disability-Adjusted Life Years) | Solomon Islands | 4.1 (0.3, 11.9) | 18.1 (1.3, 52.5) | 3.4 (0.3, 9.8) | 6.4 (0.5, 18.1) | -4.4 (-4.9,-3.9) |
| DALYs (Disability-Adjusted Life Years) | Georgia | 5316.3 (540.7, 10887.7) | 478.6 (48.7, 980.2) | 328 (30.8, 764.1) | 31.2 (2.9, 72.8) | -11.5 (-12.9,-10.1) |
| DALYs (Disability-Adjusted Life Years) | Armenia | 372 (30.8, 828.8) | 76.4 (6.3, 170.3) | 207.8 (19.8, 495.6) | 26.4 (2.5, 63) | -4.7 (-5.6,-3.8) |
| DALYs (Disability-Adjusted Life Years) | Kazakhstan | 289.9 (28.5, 736.9) | 13.9 (1.4, 35.2) | 16.6 (0, 56.3) | 0.5 (0, 1.8) | -14.9 (-16.9,-12.8) |
| DALYs (Disability-Adjusted Life Years) | Kyrgyzstan | 357.2 (41, 813.8) | 69.4 (8, 158) | 242.6 (24.2, 591.8) | 28.7 (2.9, 70) | -3.5 (-4.7,-2.3) |
| DALYs (Disability-Adjusted Life Years) | Turkmenistan | 343 (36.7, 730.3) | 105.6 (11.3, 224.9) | 284.2 (25.1, 679.3) | 40.2 (3.5, 96) | -4.9 (-6.1,-3.8) |
| DALYs (Disability-Adjusted Life Years) | Mongolia | 147.1 (14, 331.3) | 84.9 (8.1, 191.2) | 85.3 (9.2, 220.6) | 21.6 (2.3, 55.9) | -5.7 (-6.6,-4.7) |
| DALYs (Disability-Adjusted Life Years) | Uzbekistan | 124.2 (8.3, 335.6) | 6.4 (0.4, 17.2) | 51 (0.9, 165.1) | 1.1 (0, 3.6) | -7.9 (-9.0,-6.7) |
| DALYs (Disability-Adjusted Life Years) | Tajikistan | 394.3 (40.9, 886.2) | 84.2 (8.7, 189.2) | 256.2 (26.2, 606.6) | 24.9 (2.6, 59.1) | -4.9 (-6.1,-3.7) |
| DALYs (Disability-Adjusted Life Years) | Bulgaria | 2218.5 (211.3, 5002.5) | 98 (9.3, 221) | 328.5 (29, 762.5) | 13.8 (1.2, 32.1) | -9.1 (-10.4,-7.8) |
| DALYs (Disability-Adjusted Life Years) | Croatia | 1290.5 (149.8, 2851) | 115.9 (13.5, 256.1) | 161.7 (16.4, 415.4) | 10.8 (1.1, 27.9) | -9.8 (-11.0,-8.7) |
| DALYs (Disability-Adjusted Life Years) | Albania | 270.4 (28.6, 592.2) | 77.9 (8.2, 170.7) | 162.2 (13.7, 364.2) | 20.6 (1.7, 46.3) | -5.2 (-5.9,-4.5) |
| DALYs (Disability-Adjusted Life Years) | Czechia | 11064 (1057.9, 22434.4) | 467.3 (44.7, 947.5) | 1468.1 (142.7, 3251.4) | 42 (4.1, 93.1) | -9.6 (-10.5,-8.6) |
| DALYs (Disability-Adjusted Life Years) | Bosnia and Herzegovina | 45.5 (2.9, 128.7) | 6.2 (0.4, 17.5) | 4.9 (0.1, 16.9) | 0.4 (0, 1.5) | -11.8 (-13.5,-10.1) |
| DALYs (Disability-Adjusted Life Years) | Montenegro | 87.6 (7.9, 203) | 80.4 (7.2, 186.3) | 47.6 (4.8, 108.5) | 27.3 (2.8, 62.2) | -4.7 (-5.5,-4.0) |
| DALYs (Disability-Adjusted Life Years) | Poland | 10638.8 (1009.7, 23729.3) | 138.1 (13.1, 307.9) | 1360.2 (160.4, 3208.3) | 11.2 (1.3, 26.5) | -10.5 (-11.6,-9.4) |
| DALYs (Disability-Adjusted Life Years) | Hungary | 9021.1 (884.6, 18089.5) | 350.2 (34.3, 702.3) | 0 (0, 0) | 0 (0, 0) | NA (NA,NA) |
| DALYs (Disability-Adjusted Life Years) | North Macedonia | 123.4 (12.2, 298.4) | 37.5 (3.7, 90.7) | 65.9 (6.2, 167) | 11.1 (1, 28.2) | -5.4 (-6.1,-4.7) |
| DALYs (Disability-Adjusted Life Years) | Romania | 5606.2 (632.2, 12536.4) | 112 (12.6, 250.5) | 1544 (135.2, 3758) | 25.7 (2.3, 62.6) | -6.7 (-7.5,-5.9) |
| DALYs (Disability-Adjusted Life Years) | Serbia | 1736.2 (165.9, 3899.1) | 83.5 (8, 187.5) | 414 (40.1, 978.9) | 14.8 (1.4, 34.9) | -7.9 (-9.0,-6.8) |
| DALYs (Disability-Adjusted Life Years) | Slovakia | 4427.1 (497.3, 9421.8) | 426.7 (47.9, 908.2) | 1159.1 (109.9, 2496.9) | 70.7 (6.7, 152.2) | -7.4 (-8.5,-6.3) |
| DALYs (Disability-Adjusted Life Years) | Belarus | 301.3 (22.7, 765.3) | 13 (1, 33.1) | 13.9 (0, 48.5) | 0.5 (0, 1.7) | -15.0 (-17.2,-12.7) |
| DALYs (Disability-Adjusted Life Years) | Estonia | 2881.9 (260.4, 5666) | 803.9 (72.6, 1580.6) | 476.2 (47.3, 1067.8) | 108.9 (10.8, 244.1) | -7.5 (-8.5,-6.5) |
| DALYs (Disability-Adjusted Life Years) | Slovenia | 1717.5 (149, 3412.4) | 398.8 (34.6, 792.3) | 0 (0, 0) | 0 (0, 0) | NA (NA,NA) |
| DALYs (Disability-Adjusted Life Years) | Republic of Moldova | 626.8 (66.8, 1456.8) | 81.2 (8.7, 188.7) | 561.7 (58, 1285.5) | 53.2 (5.5, 121.8) | -2.1 (-3.2,-0.9) |
| DALYs (Disability-Adjusted Life Years) | Latvia | 12761.9 (1112.5, 23625.5) | 2041.1 (177.9, 3778.6) | 0 (0, 0) | 0 (0, 0) | NA (NA,NA) |
| DALYs (Disability-Adjusted Life Years) | Russian Federation | 31317.7 (3013.8, 70382.9) | 99.2 (9.5, 223) | 6706 (691.8, 16054.2) | 15.8 (1.6, 37.8) | -8.5 (-9.8,-7.2) |
| DALYs (Disability-Adjusted Life Years) | Lithuania | 4042.8 (368.5, 8547.4) | 510.2 (46.5, 1078.6) | 0 (0, 0) | 0 (0, 0) | NA (NA,NA) |
| DALYs (Disability-Adjusted Life Years) | Brunei Darussalam | 7.8 (0.8, 17.8) | 50.1 (5.1, 113.7) | 13.4 (1.2, 31.4) | 22.4 (2, 52.5) | -2.5 (-2.8,-2.1) |
| DALYs (Disability-Adjusted Life Years) | Ukraine | 675.9 (47.8, 1881.7) | 5.4 (0.4, 15.1) | 97.2 (1.5, 345.3) | 0.7 (0, 2.5) | -10.0 (-11.6,-8.3) |
| DALYs (Disability-Adjusted Life Years) | Japan | 1858.3 (215.1, 4436.7) | 6.3 (0.7, 15) | 993.6 (107.3, 2289.7) | 1.9 (0.2, 4.4) | -4.6 (-5.0,-4.2) |
| DALYs (Disability-Adjusted Life Years) | Republic of Korea | 7707.5 (571.1, 15436.1) | 154.9 (11.5, 310.2) | 11641 (866, 23190.5) | 69.6 (5.2, 138.7) | -3.4 (-3.8,-2.9) |
| DALYs (Disability-Adjusted Life Years) | New Zealand | 4010.3 (414.3, 7600.4) | 609.5 (63, 1155.1) | 1220.4 (117.5, 2491.8) | 85.6 (8.2, 174.7) | -7.5 (-8.1,-7.0) |
| DALYs (Disability-Adjusted Life Years) | Singapore | 222.2 (26, 535.3) | 61.3 (7.2, 147.6) | 77 (7, 178.6) | 5.1 (0.5, 11.8) | -9.8 (-10.8,-8.9) |
| DALYs (Disability-Adjusted Life Years) | Cyprus | 166.2 (18.9, 350.4) | 119 (13.5, 250.8) | 61.6 (6.3, 140.3) | 17.6 (1.8, 40.2) | -7.6 (-8.3,-6.9) |
| DALYs (Disability-Adjusted Life Years) | Australia | 25151.2 (2033.5, 48027.3) | 766.4 (62, 1463.6) | 8278.4 (738.3, 16140.5) | 111.8 (10, 217.9) | -7.1 (-7.5,-6.7) |
| DALYs (Disability-Adjusted Life Years) | Andorra | 5.2 (0.6, 11.7) | 52.8 (6, 118.3) | 2.8 (0.3, 6.6) | 10.8 (1, 25.1) | -5.9 (-6.4,-5.4) |
| DALYs (Disability-Adjusted Life Years) | Denmark | 4937.4 (484, 9938.3) | 378.5 (37.1, 761.8) | 0 (0, 0) | 0 (0, 0) | NA (NA,NA) |
| DALYs (Disability-Adjusted Life Years) | Austria | 556.5 (52.7, 1269.6) | 28.6 (2.7, 65.2) | 0 (0, 0) | 0 (0, 0) | NA (NA,NA) |
| DALYs (Disability-Adjusted Life Years) | Belgium | 8648.5 (687.8, 16985.3) | 329.9 (26.2, 647.8) | 1755.7 (216, 3752.1) | 46.4 (5.7, 99.2) | -7.3 (-7.9,-6.7) |
| DALYs (Disability-Adjusted Life Years) | Finland | 2666.8 (250.9, 5609.2) | 224.6 (21.1, 472.4) | 392.5 (36.5, 871.8) | 19.5 (1.8, 43.3) | -9.7 (-10.6,-8.8) |
| DALYs (Disability-Adjusted Life Years) | Greece | 3871.5 (305.6, 8282.1) | 144.8 (11.4, 309.8) | 715.6 (71.1, 1639.8) | 19.5 (1.9, 44.6) | -8.6 (-9.6,-7.6) |
| DALYs (Disability-Adjusted Life Years) | France | 12446.1 (1320, 25022.1) | 89.4 (9.5, 179.8) | 4287.7 (385, 9396) | 19.4 (1.7, 42.5) | -6.0 (-6.5,-5.4) |
| DALYs (Disability-Adjusted Life Years) | Iceland | 58.3 (4.7, 130.4) | 122.1 (9.9, 273.4) | 0 (0, 0) | 0 (0, 0) | NA (NA,NA) |
| DALYs (Disability-Adjusted Life Years) | Ireland | 655.5 (63.8, 1531.1) | 96.3 (9.4, 224.9) | 61.8 (4.9, 148.1) | 4.7 (0.4, 11.3) | -11.7 (-12.8,-10.5) |
| DALYs (Disability-Adjusted Life Years) | Germany | 118128.6 (10399.2, 236652.2) | 563 (49.6, 1127.8) | 29303.8 (2764.1, 59017.4) | 93 (8.8, 187.3) | -6.7 (-7.3,-6.2) |
| DALYs (Disability-Adjusted Life Years) | Israel | 139.3 (13.3, 352.5) | 17.2 (1.6, 43.5) | 28.1 (2.5, 75.6) | 1.4 (0.1, 3.8) | -9.6 (-10.3,-8.8) |
| DALYs (Disability-Adjusted Life Years) | Malta | 102.2 (10.1, 223.2) | 141.1 (14, 308.1) | 31.5 (3.2, 67.3) | 20 (2, 42.7) | -7.5 (-8.1,-6.9) |
| DALYs (Disability-Adjusted Life Years) | Italy | 376.8 (32.4, 973.4) | 2.5 (0.2, 6.4) | 168.2 (13.8, 445.4) | 0.7 (0.1, 2) | -4.6 (-5.2,-4.0) |
| DALYs (Disability-Adjusted Life Years) | Netherlands | 12266.2 (1187.5, 25072.3) | 370.5 (35.9, 757.3) | 4260 (368.7, 8469.4) | 72.8 (6.3, 144.8) | -6.1 (-6.6,-5.5) |
| DALYs (Disability-Adjusted Life Years) | Luxembourg | 95.1 (10.7, 193.5) | 101.9 (11.4, 207.2) | 22 (2.2, 48.8) | 12.4 (1.3, 27.4) | -7.9 (-8.5,-7.3) |
| DALYs (Disability-Adjusted Life Years) | Norway | 1659.2 (176.8, 3611.2) | 153.2 (16.3, 333.5) | 0 (0, 0) | 0 (0, 0) | NA (NA,NA) |
| DALYs (Disability-Adjusted Life Years) | Sweden | 613.1 (65.5, 1420.3) | 25.8 (2.8, 59.8) | 77.7 (6.9, 190) | 2.3 (0.2, 5.6) | -9.2 (-9.9,-8.4) |
| DALYs (Disability-Adjusted Life Years) | Portugal | 2512.6 (231.6, 5435.2) | 103.1 (9.5, 222.9) | 386.9 (40.6, 886.9) | 9.9 (1, 22.8) | -9.5 (-10.5,-8.5) |
| DALYs (Disability-Adjusted Life Years) | Switzerland | 5584.6 (602.6, 11533.9) | 330.6 (35.7, 682.8) | 989.6 (96.2, 2086.7) | 33.8 (3.3, 71.2) | -8.7 (-9.4,-8.0) |
| DALYs (Disability-Adjusted Life Years) | Spain | 212.8 (14.4, 601.6) | 2.2 (0.2, 6.3) | 58.4 (3, 168) | 0.4 (0, 1.1) | -7.2 (-8.1,-6.3) |
| DALYs (Disability-Adjusted Life Years) | United Kingdom | 59591.5 (6735.7, 118427.8) | 401.2 (45.3, 797.3) | 13061.7 (1285.3, 26367.7) | 62.1 (6.1, 125.4) | -6.7 (-7.2,-6.2) |
| DALYs (Disability-Adjusted Life Years) | Argentina | 851.9 (70.9, 1973.5) | 15.3 (1.3, 35.5) | 521.7 (46.8, 1211.5) | 5.6 (0.5, 13) | -3.1 (-3.3,-2.9) |
| DALYs (Disability-Adjusted Life Years) | Chile | 689.1 (67, 1536.7) | 41 (4, 91.4) | 0 (0, 0) | 0 (0, 0) | NA (NA,NA) |
| DALYs (Disability-Adjusted Life Years) | Uruguay | 69.6 (6.3, 179.5) | 10.3 (0.9, 26.6) | 13 (1.1, 36.8) | 1.4 (0.1, 4.1) | -7.8 (-8.5,-7.0) |
| DALYs (Disability-Adjusted Life Years) | Canada | 53510.9 (4417.6, 99131.9) | 980.2 (80.9, 1815.9) | 0 (0, 0) | 0 (0, 0) | NA (NA,NA) |
| DALYs (Disability-Adjusted Life Years) | Antigua and Barbuda | 0.3 (0, 0.7) | 2.9 (0.2, 7.8) | 0.1 (0, 0.2) | 0.3 (0, 1.1) | -8.8 (-9.4,-8.1) |
| DALYs (Disability-Adjusted Life Years) | Bahamas | 0.7 (0, 1.8) | 2.7 (0.2, 7.2) | 0.3 (0, 0.9) | 0.4 (0, 1.2) | -7.8 (-8.6,-7.1) |
| DALYs (Disability-Adjusted Life Years) | Barbados | 1.2 (0.1, 3.3) | 2.6 (0.2, 7.1) | 0.3 (0, 0.9) | 0.3 (0, 1) | -8.5 (-9.3,-7.7) |
| DALYs (Disability-Adjusted Life Years) | United States of America | 737559.4 (69789, 1379147.8) | 1405.9 (133, 2628.8) | 0 (0, 0) | 0 (0, 0) | NA (NA,NA) |
| DALYs (Disability-Adjusted Life Years) | Cuba | 73.4 (5.8, 199.8) | 4.3 (0.3, 11.8) | 21 (0.4, 67.1) | 0.6 (0, 2) | -7.9 (-8.7,-7.2) |
| DALYs (Disability-Adjusted Life Years) | Haiti | 29.3 (2.7, 80.3) | 5.4 (0.5, 14.9) | 12.3 (0.3, 39.5) | 1 (0, 3.3) | -6.7 (-7.3,-6.0) |
| DALYs (Disability-Adjusted Life Years) | Dominica | 0.3 (0, 0.8) | 3.2 (0.3, 8.6) | 0.1 (0, 0.2) | 0.5 (0, 1.4) | -7.8 (-8.5,-7.0) |
| DALYs (Disability-Adjusted Life Years) | Grenada | 0.4 (0, 1.1) | 3.4 (0.2, 9) | 0.1 (0, 0.3) | 0.5 (0, 1.6) | -8.1 (-8.8,-7.3) |
| DALYs (Disability-Adjusted Life Years) | Dominican Republic | 9.5 (0.6, 28.4) | 1.6 (0.1, 4.6) | 7 (0.1, 22.2) | 0.4 (0, 1.3) | -5.8 (-6.7,-4.9) |
| DALYs (Disability-Adjusted Life Years) | Belize | 0.4 (0, 1.1) | 2.6 (0.2, 7) | 0.2 (0, 0.5) | 0.3 (0, 1.1) | -8.6 (-9.6,-7.6) |
| DALYs (Disability-Adjusted Life Years) | Guyana | 3.1 (0.2, 8.6) | 5 (0.3, 14) | 0.8 (0, 2.6) | 0.7 (0, 2.3) | -7.4 (-8.0,-6.7) |
| DALYs (Disability-Adjusted Life Years) | Saint Lucia | 0.4 (0, 1) | 2.6 (0.2, 7) | 0.1 (0, 0.3) | 0.2 (0, 0.6) | -9.3 (-10.0,-8.6) |
| DALYs (Disability-Adjusted Life Years) | Jamaica | 4.6 (0.3, 12.3) | 1.6 (0.1, 4.2) | 1.6 (0, 4.8) | 0.3 (0, 0.9) | -6.3 (-7.0,-5.7) |
| DALYs (Disability-Adjusted Life Years) | Saint Vincent and the Grenadines | 0.4 (0, 1.1) | 3.3 (0.3, 9.5) | 0.1 (0, 0.4) | 0.5 (0, 1.4) | -8.0 (-8.6,-7.3) |
| DALYs (Disability-Adjusted Life Years) | Suriname | 1.5 (0.1, 4.2) | 3.6 (0.2, 9.5) | 0.6 (0, 1.8) | 0.5 (0, 1.6) | -7.6 (-8.4,-6.9) |
| DALYs (Disability-Adjusted Life Years) | Trinidad and Tobago | 6.2 (0.5, 17.3) | 4.5 (0.3, 12.6) | 1.9 (0, 5.8) | 0.6 (0, 1.7) | -8.9 (-9.8,-7.9) |
| DALYs (Disability-Adjusted Life Years) | Bolivia (Plurinational State of) | 922.2 (90.3, 1936.8) | 175.9 (17.2, 369.4) | 913.1 (88.7, 2115.7) | 59.1 (5.7, 136.9) | -4.2 (-4.5,-3.8) |
| DALYs (Disability-Adjusted Life Years) | Colombia | 4734.7 (527.1, 9918.4) | 164.5 (18.3, 344.5) | 2182.5 (201.7, 4817.9) | 22.8 (2.1, 50.4) | -8.4 (-9.3,-7.4) |
| DALYs (Disability-Adjusted Life Years) | Costa Rica | 1071.3 (114.6, 2088.6) | 376.7 (40.3, 734.4) | 447.1 (36.1, 959.6) | 46.7 (3.8, 100.2) | -8.5 (-9.3,-7.6) |
| DALYs (Disability-Adjusted Life Years) | Ecuador | 2803.7 (264.2, 5316.7) | 326.1 (30.7, 618.3) | 4465.1 (452.5, 8992.7) | 161.3 (16.3, 324.9) | -3.0 (-3.7,-2.4) |
| DALYs (Disability-Adjusted Life Years) | El Salvador | 822 (84, 1704.2) | 168.1 (17.2, 348.5) | 561.5 (65.3, 1228.5) | 54.9 (6.4, 120.1) | -4.8 (-5.4,-4.2) |
| DALYs (Disability-Adjusted Life Years) | Peru | 3430.8 (295.5, 7078.5) | 174 (15, 359) | 3468.2 (263.6, 7625.3) | 62 (4.7, 136.3) | -4.3 (-4.9,-3.7) |
| DALYs (Disability-Adjusted Life Years) | Mexico | 25595.9 (2522.3, 50061.1) | 368.1 (36.3, 720) | 45274.4 (4583.3, 92094) | 210.1 (21.3, 427.3) | -2.8 (-3.3,-2.3) |
| DALYs (Disability-Adjusted Life Years) | Nicaragua | 295.3 (27.9, 622.8) | 118.8 (11.2, 250.6) | 329.8 (34.1, 702) | 40.5 (4.2, 86.2) | -4.4 (-5.1,-3.7) |
| DALYs (Disability-Adjusted Life Years) | Guatemala | 132.3 (12.4, 316.9) | 23 (2.2, 55.1) | 149.3 (15.6, 367.8) | 8.2 (0.9, 20.1) | -4.3 (-4.9,-3.6) |
| DALYs (Disability-Adjusted Life Years) | Panama | 358.5 (31.2, 778.5) | 148 (12.9, 321.3) | 235.4 (20, 539.1) | 31.7 (2.7, 72.5) | -5.9 (-6.4,-5.4) |
| DALYs (Disability-Adjusted Life Years) | Venezuela (Bolivarian Republic of) | 5902.4 (591.3, 11507.2) | 376.9 (37.8, 734.9) | 4433.5 (446.2, 9887) | 84.7 (8.5, 189) | -7.1 (-8.1,-6.0) |
| DALYs (Disability-Adjusted Life Years) | Honduras | 499.7 (50.6, 1042.6) | 149.9 (15.2, 312.8) | 892.9 (81.8, 1998) | 84.9 (7.8, 190) | -2.7 (-3.3,-2.1) |
| DALYs (Disability-Adjusted Life Years) | Paraguay | 559.1 (57.8, 1146.3) | 154.1 (15.9, 316) | 599.5 (58.1, 1284.4) | 60.6 (5.9, 129.7) | -3.7 (-4.0,-3.3) |
| DALYs (Disability-Adjusted Life Years) | Egypt | 111508.6 (10206.8, 207001.9) | 2493.8 (228.3, 4629.5) | 244377.5 (19449.7, 470118) | 2208.4 (175.8, 4248.4) | -0.1 (-0.4,0.2) |
| DALYs (Disability-Adjusted Life Years) | Iran (Islamic Republic of) | 65939.3 (6459.9, 124442.3) | 1430.4 (140.1, 2699.4) | 90128.2 (8705.3, 172416.3) | 693.8 (67, 1327.2) | -2.7 (-3.1,-2.3) |
| DALYs (Disability-Adjusted Life Years) | Algeria | 2063.1 (181.1, 4551.6) | 98.5 (8.6, 217.3) | 891.1 (88.7, 2158.8) | 14.7 (1.5, 35.5) | -8.5 (-9.7,-7.4) |
| DALYs (Disability-Adjusted Life Years) | Brazil | 30175 (3351, 63005.5) | 204.2 (22.7, 426.3) | 23435.3 (2194.4, 48744.7) | 54.1 (5.1, 112.6) | -5.1 (-5.6,-4.6) |
| DALYs (Disability-Adjusted Life Years) | Bahrain | 30 (3.2, 70.9) | 107.1 (11.5, 252.5) | 72.8 (5.6, 168.5) | 44.8 (3.4, 103.8) | -3.7 (-4.6,-2.7) |
| DALYs (Disability-Adjusted Life Years) | Iraq | 1148.5 (118.5, 2636.9) | 91 (9.4, 208.9) | 4165.4 (372.3, 9546.3) | 107.2 (9.6, 245.7) | 0.5 (-0.2,1.2) |
| DALYs (Disability-Adjusted Life Years) | Jordan | 136.8 (12.2, 319.3) | 63.6 (5.7, 148.5) | 485.3 (49.3, 1104.7) | 38.6 (3.9, 87.9) | -1.8 (-2.7,-0.8) |
| DALYs (Disability-Adjusted Life Years) | Kuwait | 60.6 (5.8, 147.7) | 66.2 (6.3, 161.6) | 215.1 (18.7, 526.4) | 46.1 (4, 112.9) | -0.6 (-1.6,0.3) |
| DALYs (Disability-Adjusted Life Years) | Lebanon | 283.5 (24.8, 688.4) | 74.8 (6.5, 181.6) | 421.3 (40.6, 916.9) | 43 (4.1, 93.6) | -1.2 (-1.7,-0.8) |
| DALYs (Disability-Adjusted Life Years) | Saudi Arabia | 354.9 (27.4, 832.6) | 39.8 (3.1, 93.5) | 0 (0, 0) | 0 (0, 0) | NA (NA,NA) |
| DALYs (Disability-Adjusted Life Years) | Morocco | 2335.4 (229.2, 5187.4) | 99.6 (9.8, 221.2) | 6895.8 (587.7, 16119.3) | 114.8 (9.8, 268.3) | 0.9 (0.2,1.5) |
| DALYs (Disability-Adjusted Life Years) | Libya | 162.2 (15.9, 367.1) | 52.1 (5.1, 118) | 640 (75.4, 1578.9) | 76.5 (9, 188.8) | 2.1 (1.2,3.0) |
| DALYs (Disability-Adjusted Life Years) | Palestine | 130 (11.8, 318.1) | 89.6 (8.1, 219.2) | 313.3 (32, 707.3) | 72.8 (7.4, 164.4) | -0.4 (-1.2,0.5) |
| DALYs (Disability-Adjusted Life Years) | Oman | 113.9 (9.2, 272.3) | 113.3 (9.2, 270.9) | 214.9 (21.8, 483.3) | 68.8 (7, 154.7) | -0.9 (-1.8,-0.0) |
| DALYs (Disability-Adjusted Life Years) | Qatar | 15.4 (1.4, 36) | 94 (8.7, 220.5) | 50 (4.4, 119.9) | 32.6 (2.9, 78.3) | -3.6 (-4.7,-2.5) |
| DALYs (Disability-Adjusted Life Years) | Yemen | 902.8 (67.9, 2227.6) | 112.6 (8.5, 277.8) | 2603.4 (263.5, 6054.5) | 115.8 (11.7, 269.2) | 0.3 (-0.3,1.0) |
| DALYs (Disability-Adjusted Life Years) | Syrian Arab Republic | 996.6 (96.2, 2109.3) | 113 (10.9, 239.2) | 3135.3 (321.2, 7291) | 132.7 (13.6, 308.6) | 0.5 (-0.0,1.1) |
| DALYs (Disability-Adjusted Life Years) | Tunisia | 87.2 (7.8, 215.5) | 10 (0.9, 24.7) | 46.1 (2.5, 141.6) | 2 (0.1, 6.1) | -7.5 (-8.6,-6.5) |
| DALYs (Disability-Adjusted Life Years) | Turkey | 171.6 (11.2, 522.4) | 2.9 (0.2, 8.7) | 107.7 (2.4, 333.7) | 0.7 (0, 2) | -6.3 (-7.2,-5.5) |
| DALYs (Disability-Adjusted Life Years) | Bhutan | 165.8 (13.2, 336.1) | 417.7 (33.4, 846.9) | 243.3 (23.3, 497.2) | 244.3 (23.4, 499.2) | -2.3 (-2.5,-2.0) |
| DALYs (Disability-Adjusted Life Years) | United Arab Emirates | 85.2 (8.3, 197.8) | 151.9 (14.8, 352.5) | 220.7 (20.1, 519.2) | 30.7 (2.8, 72.3) | -5.1 (-5.5,-4.6) |
| DALYs (Disability-Adjusted Life Years) | Nepal | 7854.2 (712.7, 15678.4) | 510.2 (46.3, 1018.5) | 11664.1 (1037.2, 24187.3) | 294.7 (26.2, 611.1) | -2.1 (-2.2,-1.9) |
| DALYs (Disability-Adjusted Life Years) | India | 466897.3 (50515.9, 914973.5) | 609 (65.9, 1193.4) | 689409.2 (65152.1, 1400211.3) | 342.9 (32.4, 696.5) | -2.6 (-3.0,-2.2) |
| DALYs (Disability-Adjusted Life Years) | Pakistan | 42384.5 (5163.6, 86161.3) | 464.8 (56.6, 944.8) | 82825.9 (8078.8, 179358.3) | 417.8 (40.7, 904.7) | -0.9 (-1.3,-0.6) |
| DALYs (Disability-Adjusted Life Years) | Afghanistan | 1608.6 (177.4, 4035.4) | 132.4 (14.6, 332.1) | 1585.4 (152.7, 3624.6) | 129 (12.4, 295) | 0.2 (-0.4,0.9) |
| DALYs (Disability-Adjusted Life Years) | Bangladesh | 37617.6 (3085.8, 75032) | 496.6 (40.7, 990.6) | 56494.1 (5379.1, 116574.1) | 241.8 (23, 498.9) | -2.7 (-3.0,-2.4) |
| DALYs (Disability-Adjusted Life Years) | Angola | 30.3 (2.1, 88.4) | 4.9 (0.3, 14.2) | 37.8 (1.9, 111.2) | 2 (0.1, 5.8) | -4.1 (-4.6,-3.5) |
| DALYs (Disability-Adjusted Life Years) | Central African Republic | 12.2 (0.9, 35.2) | 6.4 (0.5, 18.6) | 8.3 (0.5, 23.7) | 2.3 (0.1, 6.6) | -4.3 (-4.8,-3.7) |
| DALYs (Disability-Adjusted Life Years) | Democratic Republic of the Congo | 125.2 (10.9, 364.2) | 4.7 (0.4, 13.8) | 108.4 (6.1, 299.6) | 1.8 (0.1, 5) | -4.3 (-4.8,-3.7) |
| DALYs (Disability-Adjusted Life Years) | Congo | 12.5 (0.7, 32.7) | 7 (0.4, 18.4) | 11 (0.7, 33.6) | 2.5 (0.2, 7.7) | -4.5 (-5.1,-3.9) |
| DALYs (Disability-Adjusted Life Years) | Equatorial Guinea | 2 (0.1, 5.6) | 6.2 (0.4, 17.4) | 1.8 (0.1, 5.4) | 2.3 (0.2, 6.8) | -4.4 (-5.0,-3.9) |
| DALYs (Disability-Adjusted Life Years) | Gabon | 5.2 (0.4, 14.4) | 5.3 (0.5, 14.7) | 3.6 (0.2, 10.8) | 2 (0.1, 6) | -4.1 (-4.8,-3.5) |
| DALYs (Disability-Adjusted Life Years) | Eritrea | 5.5 (0.4, 15.9) | 3.1 (0.2, 9) | 6.3 (0.3, 18.7) | 1.4 (0.1, 4.2) | -3.5 (-4.1,-2.9) |
| DALYs (Disability-Adjusted Life Years) | Burundi | 16 (1.4, 43.7) | 4.3 (0.4, 11.6) | 11.6 (0.6, 36.3) | 1.4 (0.1, 4.5) | -5.1 (-5.7,-4.4) |
| DALYs (Disability-Adjusted Life Years) | Kenya | 35.2 (3.6, 87.2) | 2.6 (0.3, 6.5) | 71.1 (6.4, 191.5) | 1.9 (0.2, 5.1) | -1.6 (-2.3,-1.0) |
| DALYs (Disability-Adjusted Life Years) | Djibouti | 0.5 (0, 1.6) | 2.5 (0.2, 7.4) | 1.5 (0.1, 4.5) | 1.5 (0.1, 4.4) | -2.9 (-3.5,-2.2) |
| DALYs (Disability-Adjusted Life Years) | Ethiopia | 144.4 (13.8, 382.7) | 4.5 (0.4, 11.9) | 97.9 (7, 267.3) | 1.4 (0.1, 3.9) | -4.8 (-5.3,-4.3) |
| DALYs (Disability-Adjusted Life Years) | Comoros | 1 (0.1, 2.6) | 3 (0.3, 8) | 1 (0.1, 2.7) | 1.2 (0.1, 3.3) | -4.0 (-4.5,-3.4) |
| DALYs (Disability-Adjusted Life Years) | Mozambique | 12.6 (1, 34.3) | 1.3 (0.1, 3.5) | 14.6 (0.8, 43.6) | 0.8 (0, 2.4) | -2.1 (-2.7,-1.6) |
| DALYs (Disability-Adjusted Life Years) | Rwanda | 17.6 (1.3, 49.6) | 3.8 (0.3, 10.6) | 10.9 (0.5, 29.4) | 1 (0, 2.8) | -6.1 (-6.9,-5.4) |
| DALYs (Disability-Adjusted Life Years) | Madagascar | 27.4 (2.1, 78.3) | 3.2 (0.2, 9.3) | 29 (1.5, 95) | 1.6 (0.1, 5.1) | -3.6 (-4.3,-3.0) |
| DALYs (Disability-Adjusted Life Years) | Malawi | 17.1 (1.4, 47.3) | 2.7 (0.2, 7.5) | 16.9 (1.1, 50.8) | 1.4 (0.1, 4.3) | -3.3 (-4.1,-2.5) |
| DALYs (Disability-Adjusted Life Years) | Mauritius | 10 (0.6, 27.3) | 8.2 (0.5, 22.4) | 3.6 (0.2, 10.4) | 1.1 (0, 3.1) | -8.5 (-9.4,-7.6) |
| DALYs (Disability-Adjusted Life Years) | Seychelles | 0.5 (0, 1.3) | 5.1 (0.5, 13.9) | 0.2 (0, 0.5) | 0.9 (0.1, 2.5) | -6.7 (-7.3,-6.1) |
| DALYs (Disability-Adjusted Life Years) | Uganda | 29.2 (3, 82.2) | 2.8 (0.3, 7.9) | 28.4 (1.7, 91) | 1.2 (0.1, 3.8) | -4.3 (-5.1,-3.4) |
| DALYs (Disability-Adjusted Life Years) | Zambia | 11.1 (1, 29.1) | 2.4 (0.2, 6.3) | 16.4 (0.8, 47.5) | 1.5 (0.1, 4.4) | -2.5 (-3.0,-1.9) |
| DALYs (Disability-Adjusted Life Years) | Botswana | 0.4 (0, 1.3) | 0.4 (0, 1.4) | 0.2 (0, 0.9) | 0.1 (0, 0.4) | -6.5 (-7.3,-5.7) |
| DALYs (Disability-Adjusted Life Years) | Somalia | 9.5 (0.6, 28.4) | 2.6 (0.2, 7.9) | 11.1 (0.5, 34.4) | 1.2 (0, 3.6) | -3.4 (-4.0,-2.9) |
| DALYs (Disability-Adjusted Life Years) | United Republic of Tanzania | 48.7 (3.2, 133.1) | 2.7 (0.2, 7.4) | 67.9 (3.8, 226.5) | 1.6 (0.1, 5.5) | -2.7 (-3.3,-2.1) |
| DALYs (Disability-Adjusted Life Years) | Namibia | 0.5 (0, 1.4) | 0.4 (0, 1.3) | 0.3 (0, 1) | 0.1 (0, 0.4) | -5.9 (-6.8,-4.9) |
| DALYs (Disability-Adjusted Life Years) | Lesotho | 0.3 (0, 1.2) | 0.2 (0, 0.9) | 0.2 (0, 0.7) | 0.1 (0, 0.4) | -3.4 (-4.2,-2.5) |
| DALYs (Disability-Adjusted Life Years) | South Africa | 15.7 (0.9, 44.8) | 0.5 (0, 1.3) | 0 (0, 0) | 0 (0, 0) | NA (NA,NA) |
| DALYs (Disability-Adjusted Life Years) | Eswatini | 0.2 (0, 0.7) | 0.4 (0, 1.5) | 0.1 (0, 0.5) | 0.1 (0, 0.5) | -4.7 (-5.6,-3.8) |
| DALYs (Disability-Adjusted Life Years) | Burkina Faso | 23.3 (1.4, 67.3) | 3.2 (0.2, 9.3) | 24.8 (1.2, 70.8) | 1.7 (0.1, 4.7) | -2.9 (-3.4,-2.3) |
| DALYs (Disability-Adjusted Life Years) | Cameroon | 21.5 (1.9, 59.8) | 2.9 (0.3, 8.1) | 33.6 (2.1, 92.8) | 1.7 (0.1, 4.6) | -2.7 (-3.7,-1.8) |
| DALYs (Disability-Adjusted Life Years) | Zimbabwe | 2.2 (0, 7.7) | 0.3 (0, 1.2) | 1.5 (0, 5.4) | 0.1 (0, 0.5) | -4.1 (-5.1,-3.1) |
| DALYs (Disability-Adjusted Life Years) | Cabo Verde | 1.2 (0.1, 2.9) | 2.9 (0.3, 7.4) | 1.5 (0.1, 3.9) | 2 (0.1, 5.1) | -2.7 (-3.6,-1.8) |
| DALYs (Disability-Adjusted Life Years) | Benin | 9.5 (0.9, 24.3) | 3 (0.3, 7.6) | 10.5 (0.7, 28.7) | 1.3 (0.1, 3.5) | -3.7 (-4.1,-3.2) |
| DALYs (Disability-Adjusted Life Years) | Chad | 16.3 (1.1, 41.6) | 3.5 (0.2, 9) | 16.4 (0.8, 47.4) | 1.8 (0.1, 5.1) | -3.3 (-4.0,-2.6) |
| DALYs (Disability-Adjusted Life Years) | Gambia | 2.4 (0.2, 6.5) | 4.4 (0.3, 11.8) | 3.7 (0.2, 11.4) | 2.4 (0.1, 7.4) | -2.9 (-3.6,-2.3) |
| DALYs (Disability-Adjusted Life Years) | Ghana | 51.9 (5.6, 127.6) | 5.1 (0.5, 12.5) | 42.9 (2.1, 125.7) | 1.6 (0.1, 4.6) | -5.1 (-5.8,-4.3) |
| DALYs (Disability-Adjusted Life Years) | Côte d'Ivoire | 26.7 (1.8, 73.6) | 4.2 (0.3, 11.5) | 33 (2, 102.9) | 1.8 (0.1, 5.7) | -3.8 (-4.5,-3.0) |
| DALYs (Disability-Adjusted Life Years) | Mali | 18.5 (1.5, 53) | 2.8 (0.2, 7.9) | 16.9 (1.1, 47.4) | 1.2 (0.1, 3.3) | -3.5 (-4.0,-3.1) |
| DALYs (Disability-Adjusted Life Years) | Guinea | 20.2 (1.5, 52.1) | 3.7 (0.3, 9.4) | 17.5 (1.4, 51.1) | 1.9 (0.2, 5.6) | -2.9 (-3.5,-2.3) |
| DALYs (Disability-Adjusted Life Years) | Mauritania | 9 (0.7, 24.7) | 5.4 (0.4, 14.9) | 6.8 (0.5, 19.1) | 1.9 (0.1, 5.3) | -4.7 (-5.3,-4.1) |
| DALYs (Disability-Adjusted Life Years) | Guinea-Bissau | 3.9 (0.3, 10.8) | 6.1 (0.5, 16.9) | 3 (0.2, 10) | 2.6 (0.1, 8.7) | -3.7 (-4.3,-3.1) |
| DALYs (Disability-Adjusted Life Years) | Niger | 10.8 (1, 28.1) | 2.4 (0.2, 6.4) | 16.3 (1, 45.6) | 1.2 (0.1, 3.3) | -3.3 (-3.9,-2.8) |
| DALYs (Disability-Adjusted Life Years) | Liberia | 7.9 (0.7, 21.8) | 4.2 (0.4, 11.6) | 6 (0.4, 17.5) | 1.8 (0.1, 5.3) | -3.8 (-4.4,-3.1) |
| DALYs (Disability-Adjusted Life Years) | Sierra Leone | 17 (1.4, 43) | 5.1 (0.4, 13) | 13.4 (0.7, 43.5) | 2.2 (0.1, 7.2) | -3.5 (-4.2,-2.9) |
| DALYs (Disability-Adjusted Life Years) | Nigeria | 404.9 (26.9, 1006.5) | 5.6 (0.4, 13.8) | 376.7 (37.4, 948.7) | 2.5 (0.2, 6.3) | -3.3 (-3.8,-2.7) |
| DALYs (Disability-Adjusted Life Years) | Togo | 8.3 (0.7, 22.3) | 4.2 (0.3, 11.4) | 11.5 (0.6, 37.8) | 1.8 (0.1, 6) | -3.9 (-4.6,-3.2) |
| DALYs (Disability-Adjusted Life Years) | Sao Tome and Principe | 0.3 (0, 0.9) | 3 (0.3, 7.7) | 0.3 (0, 0.8) | 1.7 (0.1, 4.6) | -2.8 (-3.5,-2.1) |
| DALYs (Disability-Adjusted Life Years) | Senegal | 26.1 (2.3, 73) | 4.9 (0.4, 13.8) | 25.8 (2.1, 71.4) | 2 (0.2, 5.5) | -4.0 (-4.6,-3.4) |
| DALYs (Disability-Adjusted Life Years) | American Samoa | 0.4 (0, 0.9) | 9.7 (0.9, 25.8) | 0.3 (0, 1) | 3.9 (0.3, 11.7) | -3.9 (-4.4,-3.3) |
| DALYs (Disability-Adjusted Life Years) | Bermuda | 0.5 (0, 1.3) | 4.9 (0.4, 12.5) | 0.1 (0, 0.3) | 0.4 (0, 1.3) | -9.4 (-10.1,-8.7) |
| DALYs (Disability-Adjusted Life Years) | Guam | 1.4 (0.1, 3.8) | 11 (0.9, 29.8) | 1.3 (0.1, 3.7) | 3.4 (0.3, 9.9) | -4.4 (-4.8,-3.9) |
| DALYs (Disability-Adjusted Life Years) | Cook Islands | 0.2 (0, 0.5) | 9.6 (0.8, 24.9) | 0.1 (0, 0.3) | 2.5 (0.2, 6.7) | -5.3 (-5.9,-4.8) |
| DALYs (Disability-Adjusted Life Years) | Niue | 0.1 (0, 0.1) | 16.5 (1.6, 40.6) | 0 (0, 0.1) | 5.9 (0.4, 15.9) | -4.4 (-4.9,-3.9) |
| DALYs (Disability-Adjusted Life Years) | Northern Mariana Islands | 0.2 (0, 0.5) | 7.9 (0.6, 21.7) | 0.3 (0, 1) | 3.2 (0.2, 10.2) | -3.8 (-4.2,-3.3) |
| DALYs (Disability-Adjusted Life Years) | Monaco | 8.8 (0.8, 19) | 79.1 (7, 171.2) | 2.2 (0.2, 5.1) | 14.2 (1.4, 32.8) | -6.7 (-7.2,-6.2) |
| DALYs (Disability-Adjusted Life Years) | Greenland | 55.5 (4.7, 108.2) | 1007.8 (85.8, 1963.6) | 52.5 (4.3, 102.8) | 387.1 (31.8, 757) | -3.1 (-3.3,-2.9) |
| DALYs (Disability-Adjusted Life Years) | Nauru | 0.2 (0, 0.5) | 24.9 (2.2, 68.9) | 0.1 (0, 0.3) | 9.7 (0.5, 30.4) | -4.3 (-5.0,-3.5) |
| DALYs (Disability-Adjusted Life Years) | Palau | 0.2 (0, 0.6) | 14.9 (1.3, 38.3) | 0.2 (0, 0.5) | 4.6 (0.3, 12.7) | -4.9 (-5.4,-4.3) |
| DALYs (Disability-Adjusted Life Years) | Puerto Rico | 19.5 (1.6, 52.2) | 3.2 (0.3, 8.6) | 4.1 (0.1, 12) | 0.3 (0, 1) | -9.0 (-9.8,-8.1) |
| DALYs (Disability-Adjusted Life Years) | San Marino | 3.1 (0.3, 6.5) | 52.3 (5, 111.1) | 0.9 (0.1, 2) | 7.4 (0.8, 17) | -6.7 (-7.3,-6.1) |
| DALYs (Disability-Adjusted Life Years) | Tokelau | 0 (0, 0.1) | 14.6 (1.2, 36.4) | 0 (0, 0) | 5.1 (0.4, 14) | -4.4 (-5.0,-3.9) |
| DALYs (Disability-Adjusted Life Years) | Saint Kitts and Nevis | 0.3 (0, 0.8) | 4.8 (0.4, 12.8) | 0.1 (0, 0.2) | 0.4 (0, 1.4) | -9.3 (-10.2,-8.4) |
| DALYs (Disability-Adjusted Life Years) | Tuvalu | 0.2 (0, 0.5) | 17 (1.3, 45.4) | 0.1 (0, 0.3) | 5.9 (0.5, 16.9) | -4.5 (-5.1,-3.9) |
| DALYs (Disability-Adjusted Life Years) | South Sudan | 12.7 (1.3, 37.1) | 3.1 (0.3, 8.9) | 8.9 (0.5, 24.7) | 1.4 (0.1, 4) | -3.6 (-4.2,-3.1) |
| DALYs (Disability-Adjusted Life Years) | United States Virgin Islands | 0.5 (0, 1.4) | 3.7 (0.2, 10.3) | 0.2 (0, 0.5) | 0.5 (0, 1.6) | -7.7 (-8.5,-7.0) |
| DALYs (Disability-Adjusted Life Years) | Sudan | 1747.2 (202.5, 3925.7) | 117.5 (13.6, 264) | 3519.8 (328.1, 8421.6) | 113 (10.5, 270.5) | 0.1 (-0.5,0.8) |
